# A conserved small RNA-generating gene cluster undergoes sequence diversification and contributes to plant immunity

**DOI:** 10.1038/s41467-026-77164-1

**Published:** 2026-08-26

**Authors:** Li Feng, Yingnan Hou, AmirAli Toghani, Zhixue Wang, Bozeng Tang, Nicola Atkinson, Hui Li, Yue Qiao, Yan Wang, Jian Hua, Jixian Zhai, Wenbo Ma

**Affiliations:** 1 The Sainsbury Laboratory, University of East Anglia, Norwich, UK; 2 Shanghai Collaborative Innovation Center of Agri-Seeds/School of Agriculture & Biology, Shanghai Jiao Tong University, Shanghai, China; 3 School of Integrative Plant Science Plant Biology Section, Cornell University, Ithaca, NY USA; 4 Department of Biological Science, University of Toronto, Scarborough, Toronto, ON Canada; 5 Institute of Plant and Food Science, Department of Biology, Southern University of Science and Technology, Shenzhen, China

**Keywords:** Plant immunity, Plant sciences

## Abstract

Small RNA-mediated gene silencing contributes to plant immunity. The secondary small interfering RNA (siRNA) pathway promotes defense by silencing target genes in invading fungal and oomycete pathogens. Many secondary siRNAs are derived from transcripts potentially encoding pentatricopeptide repeat (PPR) proteins. Here, we report that siRNA production is an ancient function of a conserved clade of *PPR* genes that undergo extensive within-species diversification. In *Arabidopsis thaliana*, siRNA-source *PPR*s are physically clustered on Chromosome 1. These sequences are diversified through gene duplication followed by sequence diversification and accumulation of high-impact variations including pseudogenization, leading to the accumulation of a diverse siRNA pool. These features are consistent with the engagement of *PPR*-siRNAs in a co-evolutionary arms race with the pathogens. This study defines siRNA-producing *PPR*s as a class of defense genes and highlights the potential of *PPR*-siRNA-based engineering as a strategy to enhance disease resistance.

## Abstract (web summary)

Here the authors reveal a conserved small RNA-producing gene cluster that undergoes sequence diversification, driving a co-evolutionary arms race with pathogens. This native innate immunity offers new opportunities to engineer disease resistance in plants.

## Introduction

Plants employ a sophisticated immune system to combat pathogens. Pathogen perception activates immune signaling that leads to various defense responses such as cell wall reinforcement, reactive oxygen species (ROS) production, and release of antimicrobial molecules^1^. Recent studies suggest that small RNAs (sRNAs) can function as antimicrobial agents by directly targeting pathogen genes through a process termed host-induced gene silencing (HIGS)^2,3^.

sRNAs are short, non-coding RNA molecules that repress target gene expression through nucleotide base pairing^4^. Plant sRNAs are classified into two major categories, microRNAs (miRNAs) and small-interfering RNAs (siRNAs)^5^. miRNAs originate from *MIRNA* (*MIR*) genes, whose primary transcripts fold into imperfectly paired stem-loop structures^6^. siRNAs, on the other hand, arise from perfectly matched double-stranded RNAs (dsRNAs) generated by RNA-dependent RNA polymerases (RDRs)^7^. Dicer-like (DCL) proteins process *MIR* stem-loops or RDR-derived dsRNA precursors into miRNA or siRNA duplexes, which are subsequently loaded into ARGONAUTE (AGO) protein complexes for target gene silencing^6,8^. Some siRNAs require cleavage of specific transcripts guided by miRNAs to initiate their biogenesis and are thereby named secondary siRNAs. The cleaved RNAs are then used as templates to produce dsRNA precursors, which are further processed into an array of 21 or 24 nt siRNA duplexes^9^. These “secondary” siRNAs are spaced in phased intervals and are therefore also called phased siRNAs or phasiRNAs. Sequences that give rise to phasiRNAs include the non-coding *TAS* genes and protein-coding genes, especially those predicted to encode Nucleotide-binding leucine-rich repeat domain (NLR) and pentatricopeptide repeat (PPR) proteins^10^.

The functional significance of the secondary siRNA pathway lies in the potential widespread gene silencing through a cascade in which secondary siRNAs could target genes not regulated by the primary miRNAs, hence amplifying the silencing efficacy. This is believed to be particularly effective in regulating large gene families, such as NLRs and PPRs^9^. In particular, NLRs are canonical immune receptor proteins and siRNAs produced through the miRNA-*NLR*-siRNA and/or miRNA-*TAS*-*NLR*-siRNA pathways potentially have a global impact on *NLR* gene expression^10–13^. For example, in soybean and potato, members of the miR482/miR2118 superfamily trigger production of secondary siRNAs that presume to potentially silence a large number of *NLR* genes to prevent fitness-reducing autoimmunity in the absence of pathogen attack^14^. Reduced accumulation of miR482/miR2118b in tomato led to enhanced resistance to the oomycete pathogen *Phytophthora infestans* and the bacterial pathogen *Pseudomonas syringae*, likely due to decreased production of *NLR*-silencing siRNAs and the subsequent increase of NLR expression^12^. More recently, *trans*-acting siRNAs (tasiRNAs) generated from *TAS3* were found to regulate systemically acquired resistance by modulating the expression of auxin response factors^15^.

In addition to regulating endogenous gene expression, secondary siRNAs have been found to elevate defense by silencing genes invading eukaryotic pathogens through HIGS^3^. In *Arabidopsis thaliana*, the accumulation of siRNAs derived from *PPR* transcripts increases during the infection of the oomycete pathogen *Phytophthora capsici*^16^. Mutations in miR161, which targets a small subset of *PPR* transcripts and triggers siRNA production, lead to hypersusceptibility to *P. capsici*, suggesting a contribution of *PPR*-derived siRNAs (*PPR*-siRNAs) to defense. *A. thaliana PPR*-siRNAs represent a diverse pool with thousands of sequences that are predicted to target many *P. capsici* genes. Silencing of one of the predicted targets was confirmed during infection^16^. These findings support a role of *PPR*-siRNAs in plant defense. Consistent with this, oomycete pathogens encode silencing suppressors that contribute to virulence^16,17^.

*PPR*s constitute a large gene family in plants, with over 400 members per genome in most sequenced species^18^. A subset of PPRs has been functionally characterized as nucleotide-binding proteins that direct RNA processing complexes to specific transcripts in mitochondria and chloroplast for cleavage, thereby inhibiting their translation^18–20^. Among these PPRs, Restorer-of-Fertility (Rf) targets mitochondrial genes responsible for cytoplasmic male sterility (CMS)^21,22^. The interaction between Rf and CMS loci exemplifies a co-evolution in which mitochondrial gene mutations promoting maternal transmission via CMS are counteracted by regulators encoded in the nuclear genome^23^. This genetic conflict drives positive selection at the region of Rf and Rf-like (RFL) proteins that determines the RNA-binding specificity, leading to functional diversification^24–26^.

Recently, it was discovered that some *PPR* genes, including several *RFL* genes, can be recruited to the miRNA-triggered secondary siRNA pathway and this phenomenon is prevalent in eudicots^27^. The role of *PPR*-siRNAs in plant defense suggests that the siRNA-source loci should be engaged in a co-evolutionary arms race with the pathogens, thus may exhibit a distinct evolutionary trajectory^28^. Interestingly, most siRNA-producing genes in the *A. thaliana* accession Col-0 cluster together and distantly from other *PPR* genes in a phylogenetic tree generated from ~300 PPR proteins^29^. Whether this pattern is common in *A. thaliana* and other plants, and how they are correlated with siRNA production, remains to be explored.

In this study, we systematically analyzed siRNA-producing *PPR* genes in eight *A. thaliana* accessions and expanded this analysis to a wide range of evolutionarily diverse plant species. In *A. thaliana*, we found that siRNA-producing *PPR*s are physically and phylogenetically clustered. The siRNA-producing *PPR*s, but not those that do not give rise to siRNAs, exhibit a high level of sequence diversity, consistent with their engagement in host-pathogen co-evolution. Despite the sequence variation, siRNA-producing *PPR*s retain the target sites of miR161 and several tasiRNAs that can trigger secondary siRNA production, resulting in a diverse pool of *PPR*-siRNAs. Functional analysis supports the hypothesis that siRNA production, not putative protein products, is responsible for the disease resistance function of a siRNA-source *PPR* gene. Importantly, PPR analysis of evolutionarily diverse plant species revealed a dedicated “siRNA-producing” clade that exhibits within-species diversification, suggesting that siRNA production is an ancient function of a specific phylogenetic group of *PPR* genes. Finally, we show that heterologous expression of an *A. thaliana PPR*-siRNA-producing cassette can enhance *Phytophthora* resistance in *Nicotiana benthamiana*. This study defines siRNA-producing *PPR*s as a family of defense genes and provides a proof-of-concept strategy to elevate disease resistance by engineering *PPR*-siRNAs.

## Results

### A dedicated siRNA-producing *PPR* gene cluster in *A. thaliana*

We investigated *PPR*-siRNA populations and siRNA-producing *PPR* loci in eight *A. thaliana* accessions with high-quality genome assemblies, including An-1, Col-0, Ct-1, Cvi-1, Eri-1, Kyo-1, L*er*-0, and Sha^30–32^. Rosette leaves from 2-week-old plants were used to generate sRNA libraries for sequencing. After quality control and exclusion of structural RNA-derived reads, we obtained 1–7 million genome-matched reads from each accession in the size range of 18–28 nt, comprising 0.4–1 million unique reads (Supplementary Data 1). Size distribution of sRNA population in each accession exhibited two prominent peaks at 21-nt and 24-nt (Supplementary Fig. 1a), consistent with typical patterns observed in *A. thaliana*^33^.

Using this dataset, we determined *PPR* loci from which siRNAs were spawned. For this purpose, PPR-encoding genes were identified from each *A. thaliana* accession based on sequence similarity with a set of 475 *PPR* genes defined in Col-0^34^. Using SimpleSynteny^35^, BLASTN, and BLASTP^36^, 467, 471, 459, 465, 464, 467, and 461 *PPR* genes were annotated in An-1, Ct-1, Cvi-1, Eri-1, Kyo-1, L*er-*0, and Sha, respectively (Supplementary Data 2). Total siRNAs extracted from these *PPR* loci showed a predominant peak at 21-nt in all accessions (Supplementary Fig. 1b), consistent with the previous observation in Col-0^29^.

**Fig. 1:**
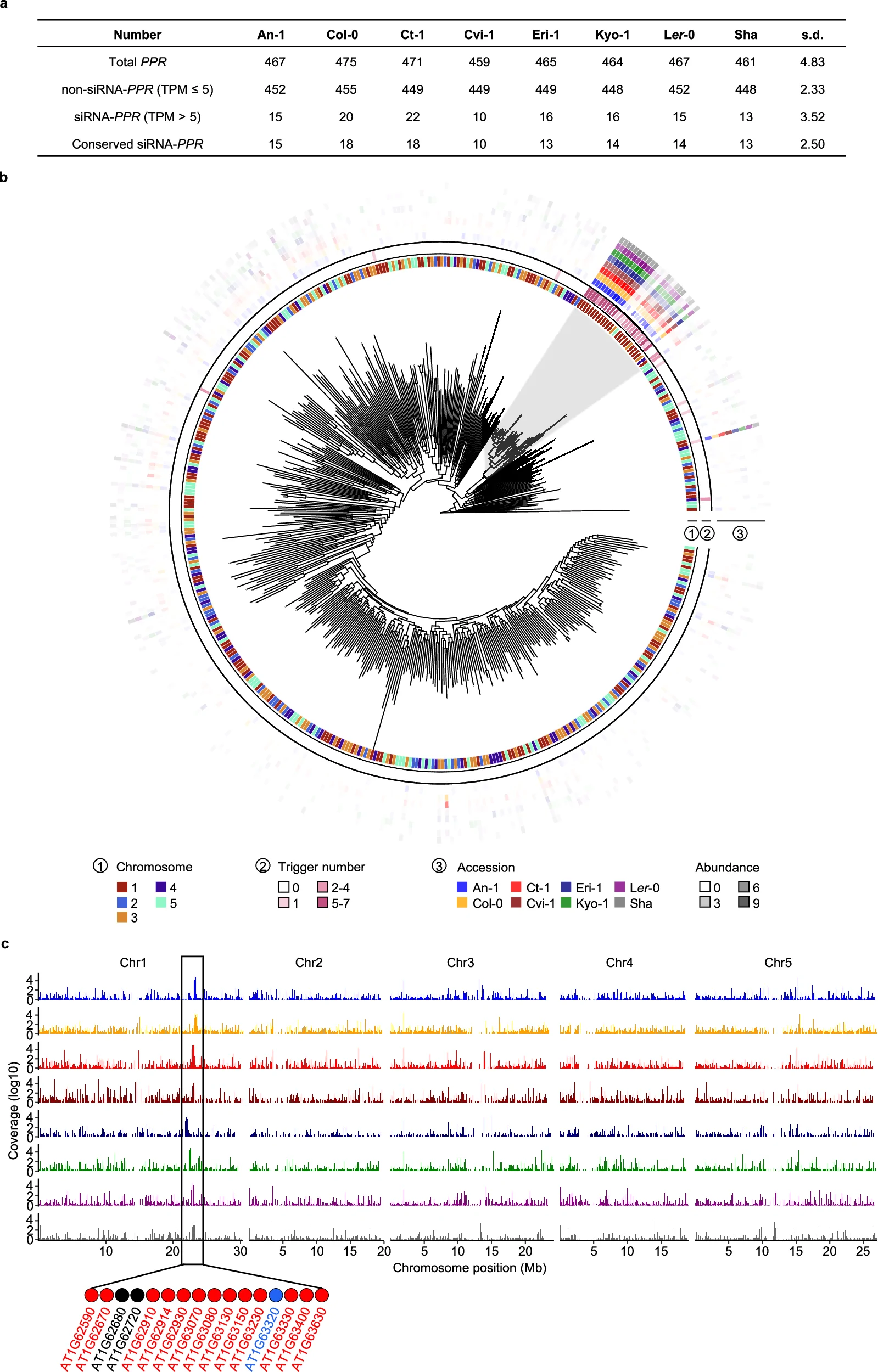
A designated *PPR* gene cluster produces siRNAs in *A. thaliana.* **a** siRNA-producing *PPR* (siRNA-*PPR*) homologs identified in eight *A. thaliana* accessions. Conserved siRNA-*PPR*s were defined as *PPR*s that accumulated siRNAs with a TPM (Transcripts Per Million) value > 5 in at least five accessions. s.d. represents the value of standard deviation. **b** A phylogenetically defined clade of *PPR*s produces siRNAs. A phylogenetic tree was constructed using the complete set of 475 PPRs in *A. thaliana* Col-0 using amino acid sequences. Ring 1 indicates the chromosomal location of each PPR-encoding gene. Ring 2 indicates the number of sRNA triggers predicted from each *PPR* transcript. These sRNA triggers include miR161.1, miR161.2, miR400, TAS1a 3′ D9 (–), TAS1b 3′ D4 (–), TAS1c 3′ D6 (–), TAS1c 3′ D10 (–), TAS2 3′ D6 (–), TAS2 3′ D9 (–), TAS2 3′ D11 (–), and TAS2 3′ D12 (–). Ring 3 presents the abundance of 21-nt siRNAs derived from each *PPR* homolog in the eight *A. thaliana* accessions. siRNA abundances are represented by log_2_-transformed TPM. Highlighted in gray is a phylogenetic group that produces the majority of *PPR*-siRNAs. **c** Physical clustering of siRNA-producing *PPR*s. The majority of *PPR*-siRNAs are produced from a conserved gene cluster on Chromosome 1. The entire complement of 21-nt siRNAs derived from coding sequences in each of the eight *A. thaliana* accessions are mapped to their chromosomal locations. A major peak is highlighted on the longer arm of Chromosome 1, representing a sRNA-producing hotspot that harbors multiple siRNA-producing *PPR* genes. Red dots label siRNA-producing *PPR*s that belong to the phylogenetic group highlighted in panel (**b**). The gene with the blue dot produces siRNAs but is not clustered within the phylogenetic group highlighted in panel (**b**). The black dots label non-siRNA-producing *PPR*s. A Source Data file is also provided.

To identify siRNA-producing *PPR*s, we calculated the normalized accumulation (transcripts per million, TPM) of siRNAs derived from each *PPR* gene in these *A. thaliana* accessions. Using a cutoff of TPM > 5, 127 *PPR* genes were identified as siRNA-producing loci (Fig. 1a; Supplementary Data 3). While the numbers of non-siRNA-producing *PPR*s remain relatively constant in all accessions (from 448 to 455, with a standard deviation of 2.33), the numbers of siRNA-producing genes are more variable (from 10 to 22, with a standard deviation of 3.52). Furthermore, for non-siRNA-producing *PPR*s, one gene in Col-0 has a single homolog in each of the other seven accessions in almost all cases. In contrast, siRNA-producing *PPR*s often have multiple homologous genes in another accession (Supplementary Fig. 1c; Supplementary Data 3). These observations indicate an increased variability in siRNA-producing *PPR*s among the accessions.

Twenty *PPR*s produced siRNAs in Col-0. Mapping these siRNA-producing *PPR*s on a phylogenetic tree generated using the amino acid sequences of the entire set of 475 PPRs revealed that 18 of the siRNA-producing *PPR*s belong to the same clade (Fig. 1b; Supplementary Data 3). This result is similar to the previous analysis using a smaller set of PPRs^29^. These 18 genes are responsible for the production of 97.95% of the total 21-nt *PPR*-siRNA population in Col-0 (Supplementary Data 3).

The initial step of secondary siRNA production involves targeting of siRNA-producing transcripts by trigger sRNAs, being miRNA or siRNAs, via sequence complementarity^37,38^. Eleven miRNAs and tasiRNAs have previously been identified as potential triggers of *PPR*-siRNA production in *A. thaliana* through the miRNA-*PPR*-siRNA or miRNA-*TAS*-*PPR*-siRNA pathways^27,29^. These trigger sRNAs include miR161.1, miR161.2, miR400, and eight tasiRNAs derived from *TAS1* and *TAS2*. Target site prediction suggests that the 18 siRNA-producing *PPR*s belonging to the same phylogenetic group are highly enriched with target sites of the 11 sRNA triggers (Fig. 1b Ring 2; Supplementary Data 4). Many gene members are predicted to contain target sites of more than five trigger sRNAs, correlated with their role as the main *PPR*-siRNA producers in the genome.

We next determined the conserved siRNA-producing *PPR*s using a criterium that siRNA production must be detectable in at least five *A. thaliana* accessions from the same homologous gene. This analysis results in the identification of 115 genes in the eight accessions that we named as “core” siRNA-producing *PPR*s (Fig. 1a; Supplementary Data 3). Seventeen of the 18 core siRNA producers in Col-0 belong to the same phylogenetic group highlighted in Fig. 1b. Remarkably, homologs of these 17 genes in the other accessions are also the most prominent siRNA producers in their specific accession, suggesting that siRNA production is a conserved feature of this phylogenetic group in *A. thaliana* (Fig. 1b Ring 3).

We noticed that all siRNA-producing *PPR*s in this phylogenetic group, except for one, are located on Chromosome 1 (Fig. 1b; magenta-colored grids in Ring 1). To further investigate how these genes may be physically linked, we conducted a genome-wide analysis and mapped the entire population of 21-nt siRNAs derived from coding sequences in each *A. thaliana* accessions. The results revealed a prominent peak on the long arm of Chromosome 1 as a major siRNA-producing hotspot in a similar location of all accessions (Fig. 1c; Supplementary Fig. 1d). In Col-0, 16 *PPR* genes are located within a region of 0.4 Mb (nucleotides 23,179,248–23,587,005), among which include 14 siRNA-producing *PPR*s (Fig. 1c; Supplementary Data 3). All of these 14 genes, except for one (AT1G63320), belong to the same phylogenetic group highlighted in Fig. 1b. siRNAs produced from this gene cluster account for 84.07–98.10% of the total *PPR*-siRNAs across the eight *A. thaliana* accessions (Supplementary Fig. 1e). Taken together, these analyses identified a phylogenetically related and physically linked *PPR* gene cluster that serves as a conserved siRNA-producing hotspot in *A. thaliana*.

### Genetic dynamics in the siRNA-producing *PPR* cluster

Physical clustering of genes belonging to the same family may accelerate their evolution. For example, genes encoding immune receptors, especially the *NLR* genes, often form clusters in the genome^39^. Some *NLR* gene clusters potentially undergo asymmetrical expansion, with certain members exhibiting a greater tendency for duplication and diversification than others^40^. We examined potential gene duplication events among 87 siRNA-producing *PPR* genes located in the siRNA-producing *PPR* gene cluster on Chromosome 1 (called “hotspot” siRNA-producing *PPR*s hereafter) by assessing their sequence similarity across the eight *A. thaliana* accessions. In addition, homologs of the siRNA-producing *PPR AT5G16640* of Col-0, which is located on Chromosome 5 but phylogenetically clustered with the hotspot siRNA-producing *PPR*s, were included in the analysis for comparison.

**Fig. 2:**
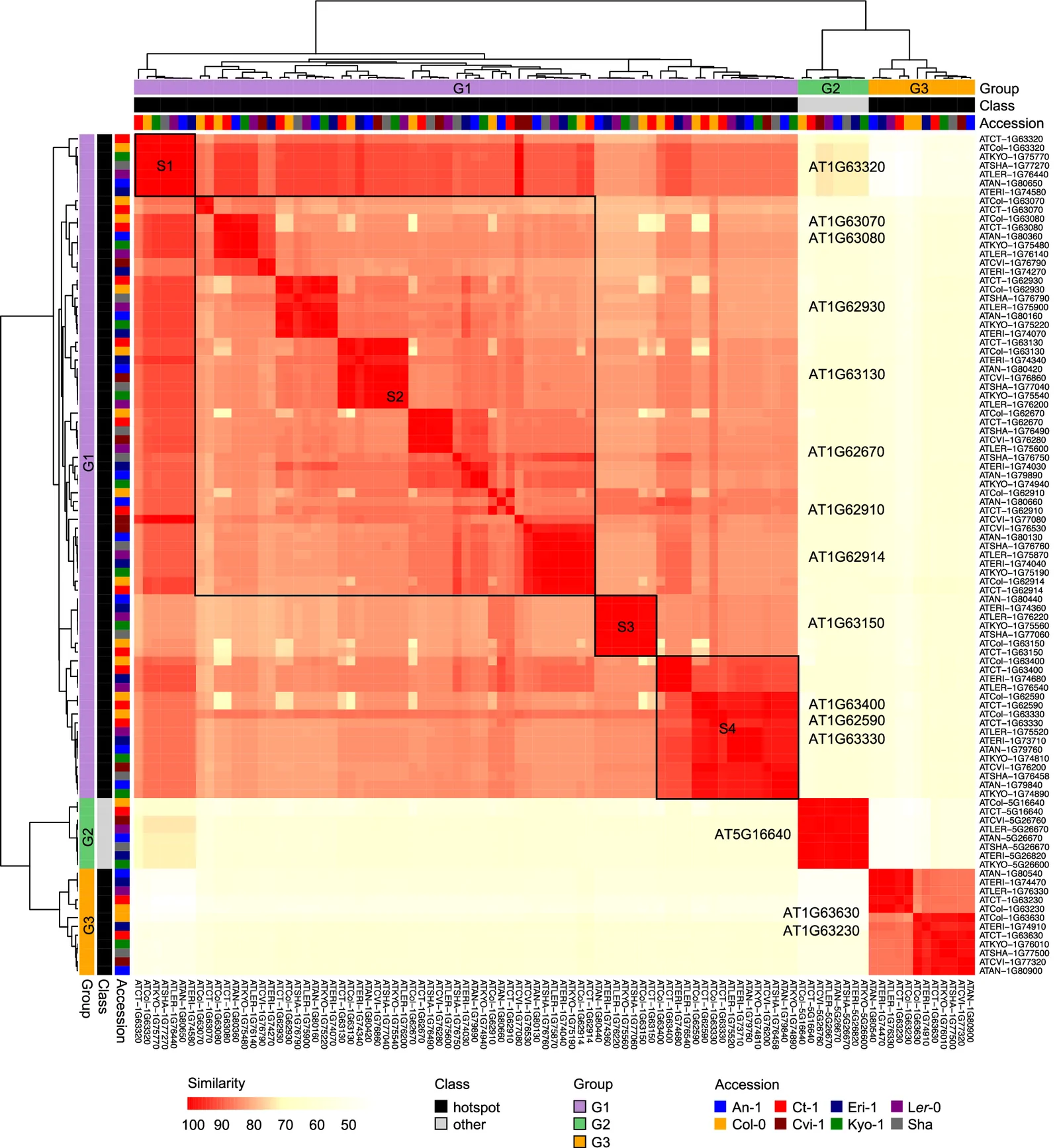
Potential gene duplication in a siRNA-producing *PPR* gene cluster of *A. thaliana.* Eighty-seven siRNA-producing *PPR* genes located in the hotspot siRNA-producing *PPR* gene cluster on Chromosome 1 were analyzed and grouped based on nucleotide sequence similarity across the eight *A. thaliana* accessions. Eight homologs of the siRNA-producing *PPR AT5G16640* (Col-0), which is located on Chromosome 5 but phylogenetically clustered with the hotspot siRNA-producing *PPR*s, were included for comparison. The hotspot siRNA-producing *PPR* gene form two groups: G1 (75 genes) and G3 (12 genes). G1 can be further grouped into four sub-groups, named S1–S4. G2 (8 genes) consists of *AT5G16640* homologs. A Source Data file is also provided.

The hotspot siRNA-producing *PPR*s can be categorized into two groups (using a cutoff of > 75% nucleotide sequence similarity) that are also clearly separated from the *AT5G16640* homologs (Fig. 2). The largest group (G1) consists of 75 genes as homologs of 12 hotspot siRNA-producing *PPR*s in Col-0. G3 consists of 12 hotspot siRNA-producing *PPR*s that are homologous to the Col-0 genes *AT1G63230* and *AT1G63630*. Compared to G1 and G3, which have variable numbers of genes in different accessions, G2 is comprised of eight homologs of the Col-0 *AT5G16640*, one in each accession (Fig. 2). The gene number variation in different accessions indicates a high degree of dynamics among the hotspot siRNA-producing *PPR*s.

Seventy-five genes in G1 share significant sequence similarity. These genes can be further classified into four sub-groups (S1–S4). Notably, homologs of the Col-0 gene *AT1G63320* form the sub-group S1, which shares a sequence similarity of > 78% with all the other genes within G1 (Fig. 2). Furthermore, *AT1G63320* contains a single intron (77 nt), which is present within the coding sequences of most other siRNA-producing *PPR*s (Fig. S2). It was previously proposed that a few intron-containing *PPR* genes in *A. thaliana* and rice may present as ancient loci that served as templates for numerous intron-less copies created during retrotransposition-driven gene expansion^41^. These intriguing observations support the possibility that duplication of *AT1G63320*-like sequences may have contributed to the expansion and diversification of siRNA-producing genes within this gene cluster.

We further investigated how the dynamics within the siRNA-producing *PPR* cluster may influence siRNA production. For this purpose, homologs of the 11 sRNAs that have already been experimentally demonstrated to trigger *PPR*-siRNA production in Col-0^29,42^ were identified in the other *A. thaliana* accessions and then used to predict target sites in *PPR*s in each accession. These sRNAs are highly conserved, with nearly 100% sequence identity, in all the eight accessions (Supplementary Fig. 3a; Supplementary Data 4). This analysis revealed distinctive patterns in siRNA-producing *PPR*s that belong to different groups with genes within one group predicted to share a similar sRNA targeting pattern (Supplementary Fig. 3b). For example, the *AT1G63320* homologs contain a predicted target site of TAS2 3′ D11 (–), which is commonly present in all the other G1 gene members but absent in members of G2 and G3. On the other hand, target sites of miR161.1, miR161.2 and TAS2 3′ D6 (–) are absent in the *AT1G63320* homologs but prevalently adopted in the other siRNA-producing *PPR*s. This is consistent with the hypothesis that *AT1G63320* may resemble an ancestral sequence from which the G1 group of siRNA-producing *PPR*s were derived to form an expanded gene cluster. The additional target sRNA sites, especially miR161 and TAS2 3′ D6 (–), may be responsible for the highly efficient production of siRNAs in these loci (Supplementary Fig. 3b). We also observed a high level of conservation in the predicted target sites of the *PPR*s, especially at the nucleotide positions that are essential for trigger sRNA-guided cleavage (Supplementary Fig. 4), providing additional support to the target site prediction.

### Sequence diversification in siRNA-producing *PPR*s

**Fig. 3:**
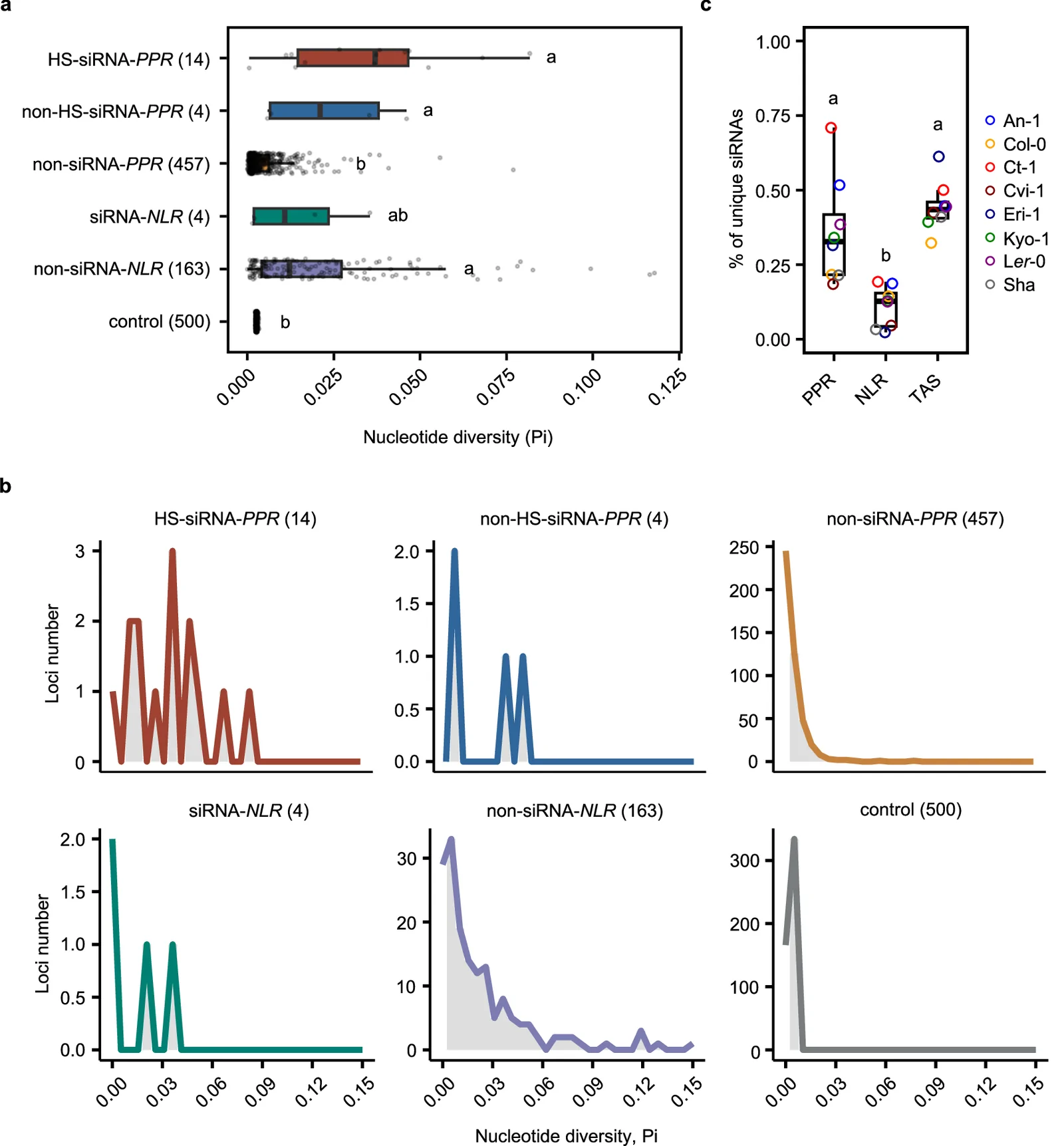
siRNA-producing *PPR*s exhibit accelerated sequence diversification in *A. thaliana.* **a** Nucleotide diversity (Pi) of *PPR* genes determined using nucleotide sequences in eight *A. thaliana* accessions. Numbers in parentheses indicate the sample size (*n* = number of genes) in each of the six gene groups: hotspot-siRNA-*PPR*s, non-hotspot-siRNA-*PPR*s, non-siRNA-*PPR*s, siRNA-*NLR*s, non-siRNA-*NLR*s, and control (randomly selected genes) in Col-0. HS, hotspot. **b** Distribution of Pi values of the six gene groups. **c**. Diversity of siRNAs derived from *PPR*, *NLR* or *TAS* transcripts. siRNA diversity was calculated as the proportion (%) of unique 21-nt siRNAs produced from each gene group in the total number of unique siRNA sequences in eight *A. thaliana* accessions. For box plots in (**a**) and (**c**), center lines represent the median (50th percentile), box limits show the 25th and 75th percentiles (interquartile range, IQR), and whiskers extend to the minima and maxima values within 1.5 x IQR. Individual points represent single genes in (**a**) and individual accessions in (**c**). Statistically significant differences among groups were evaluated using a non-parametric Kruskal-Wallis test with Bonferroni-adjusted post hoc comparisons, labeled by different letters (adjusted *P* < 0.05). Adjusted *P* values and test statistics for (**a**) and (**c**) are provided in the Source Data file.

The observations of a highly dynamic siRNA-producing *PPR* cluster prompted us to quantify nucleotide sequence diversity in these genes, which was calculated as values of Pi (average sequence divergence), Watterson’s theta (number of polymorphic sites), and genetic distance. All three measures led to the same conclusion that siRNA-producing *PPR*s exhibit substantially higher sequence diversity than non-siRNA-producing *PPR*s (Fig. 3a, b; Supplementary Fig. 5a-5b). We compared sequence diversity among hotspot siRNA-producing *PPR*s with non-hotspot siRNA-producing *PPR*s and non-siRNA-producing *PPR*s. To provide critical benchmarks and controls for these metrics, we analyzed the immune receptor *NLR* genes, which are one of the most diverse gene family in *A. thaliana*, as a positive control^39,43^. A small subset of *NLR* genes can also spawn siRNAs. Therefore, siRNA-producing and non-siRNA-producing *NLR*s were analyzed as separate groups to evaluate whether siRNA production may influence *NLR* diversification. In addition, a random set of 500 genes was used as a control group that represents a background level of sequence diversity in *A. thaliana*.

We found the control group and the non-siRNA-producing *PPR*s exhibited the lowest genetic diversity, with median Pi values of 0.0027 and 0.0023, respectively (Fig. 3a). The distribution of the Pi values also displayed a similar pattern for these two gene groups, indicating a high sequence conservation (Fig. 3b). In contrast, both hotspot siRNA-producing *PPR*s and non-hotspot-siRNA-producing *PPR*s showed a significantly higher diversity comparable to non-siRNA-producing *PPR*s, with the median Pi values of 0.037 and 0.021. The Pi values also have wider distributions, suggesting an elevated sequence diversity in the siRNA-producing *PPR*s regardless their genomic location (Fig. 3a, b). Consistent with previous studies^39,43^, *NLR* genes exhibited a significantly higher diversity than the control group, with a median Pi value similar to the siRNA-producing *PPR* genes. However, no difference was observed between the siRNA-producing and non-siRNA-producing *NLR* gene groups (Fig. 3a, b), indicating that, unlike *PPR*s, sequence diversification of *NLR*s is not driven by siRNA production. Therefore, only siRNA-producing *PPR*s showed the trend of rapid evolution under selective pressure.

Despite the diversity of siRNA-producing *PPR*s, target sites of sRNA triggers have been retained to ensure siRNA production (Supplementary Fig. 5c; Supplementary Data 4), indicating that an accelerated diversification of siRNA-producing *PPR*s may lead to diversity in *PPR*-siRNAs. To test this, we compared the sequence diversity of siRNAs spawned from *PPR* loci with those derived from *NLR* or *TAS* loci across the eight *A. thaliana* accessions. The median values of the diversity of *PPR*-siRNAs and *TAS*-siRNAs were significantly higher than that of *NLR*-siRNAs (Fig. 3c). Furthermore, the proportions of unique sequences of 21-nt siRNA originated from *PPR*, *NLR*, or *TAS* loci were calculated in each *A. thaliana* accession relative to the total number of unique 21-nt siRNAs in the same accession. Results from this analysis indicates that the overall diversity of 21-nt siRNA population is largely attributed to siRNAs derived from *PPR* and *TAS* loci (Supplementary Fig. 5d). This is consistent with a role of *PPR*- and *TAS*-siRNAs in silencing gene targets from invading pathogens, thus engaging in a host-pathogen arms race^3^.

### Contribution of high-impact variations to the diversity of siRNA-producing *PPR*s

**Fig. 4:**
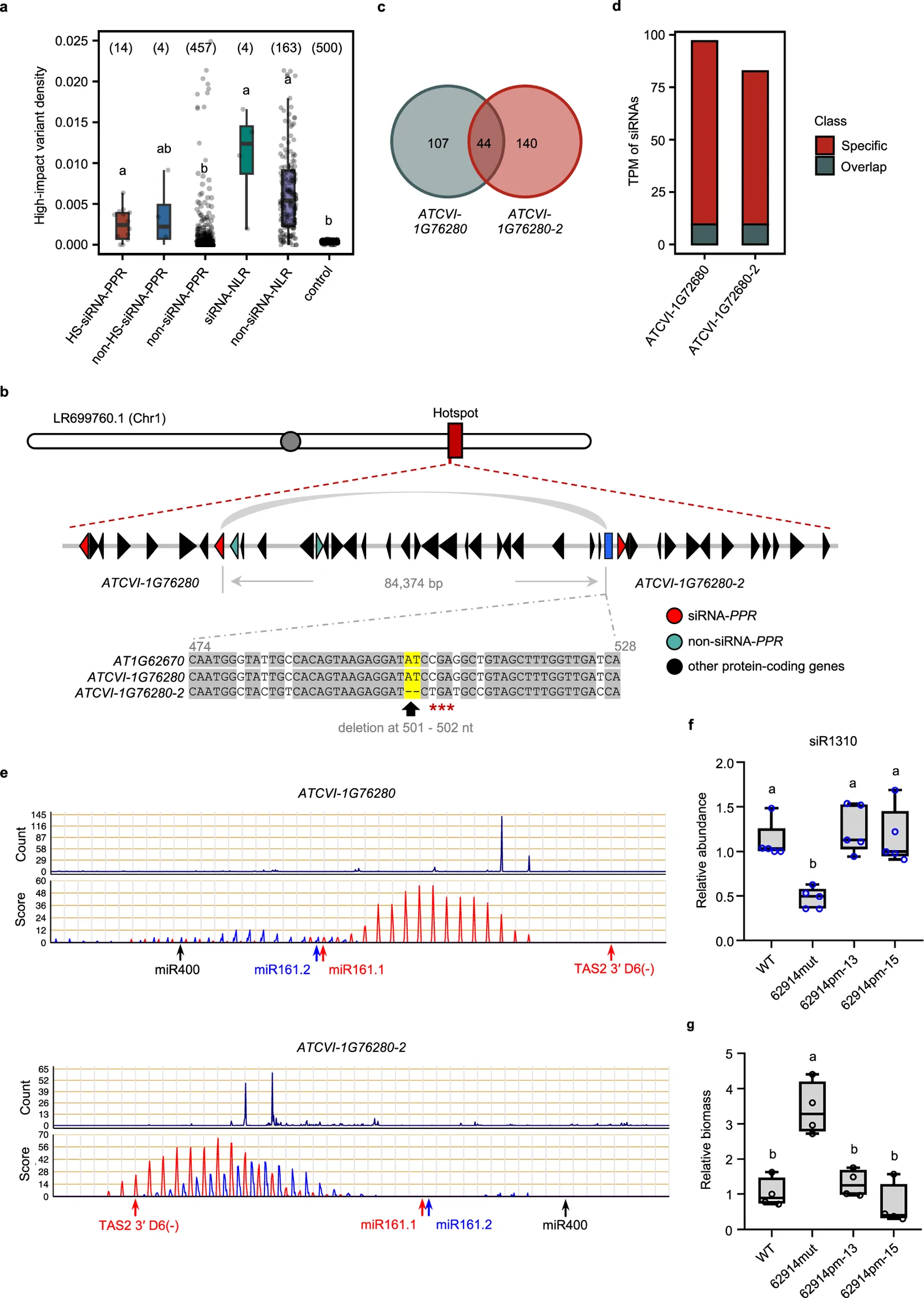
Genetic and functional consequences of high-impact variations in siRNA-producing *PPR*s. **a** Density of high-impact variants, determined as the proportion of sequence variants causing frameshift or stop codon within coding sequence, was determined in six gene groups using *A. thaliana* pangenome: hotspot-siRNA-*PPR*s, non-hotspot-siRNA-*PPR*s, non-siRNA-*PPR*s, siRNA-*NLR*s, non-siRNA-*NLR*s, and control (random genes). Numbers of genes in Col-0 are indicated. HS, hotspot. Center line, box limits, and whiskers represent median, 25th and 75th percentiles (interquartile range, IQR), and minima and maxima within 1.5 x IQR. Each point represents a single gene. Statistically significant differences were evaluated using Kruskal-Wallis test with Bonferroni-adjusted post hoc comparisons, labeled by different letters (adjusted *P* < 0.05). **b** Pseudogenization of a siRNA-producing *PPR* in Cvi-1. A region within the hotspot siRNA-producing *PPR* cluster on Chromosome 1 contains two Col-0 *PPR AT1G62670* homologs: *ATCVI-1G76280* (a coding gene, red triangle) and *ATCVI-1G76280-2* (a pseudogene, blue rectangle). Sequence alignment shows a 2-nt deletion (highlighted in yellow) in *ATCVI-1G76280*, introducing a stop codon in *ATCVI-1G76280-2*. **c**,** d** The majority of 21-nt siRNAs are uniquely produced from *ATCVI-1G76280* or *ATCVI-1G76280-2*. **e** Phasing patterns of siRNAs produced from *ATCVI-1G76280* and *ATCVI-1G76280-2*. Arrowheads indicate the predicted cleavage sites of sRNA triggers and their color (red or blue) corresponds to the phasing patterns of siRNAs, respectively. Black arrowheads indicate target sites not associated with the siRNA production. **f** Relative abundance of siR1310 in WT (Col-0), *62914mut* (a T-DNA insertion mutant of *AT1G62914*), and two independent complementation lines (*62914pm-13*, *62914pm-15*) expressing a *AT1G62914* mutant that contains a premature stop codon by introducing a T-to-A mutation at the nucleotide position 432. siRNA abundances relative to that in WT were determined by stem-loop PCR. Data are presented as mean ± SD (*n* = 5 independent biological replicates). **g** Relative biomass of *Phytophthora capsici* (mean ± SD, *n* = 4 independent biological replicates) in inoculated plants measured by qPCR at 3 days post inoculation (dpi). For (**f**) and (**g**), statistically significant differences were determined using a one-way ANOVA with Tukey’s post hoc test and labeled by different letters (*P* < 0.05). Adjusted *P* values and test statistics are provided in the Source Data file.

High-impact variations include potentially functional variants, such as sequence changes that disrupt the translational reading frame (frameshift) and/or result in a premature stop codon (stop gained)^44^. To further explore the impact of genetic variations on the diversity of siRNA-producing *PPR*s, we extended our analysis to include 1,135 *A. thaliana* genomes and detected high-impact variations from *PPR* genes. Again, *NLR* genes were included as a comparison, and a random set of 500 genes was used as the control. siRNA-producing and non-siRNA-producing *PPR* and *NLR* genes were analyzed as separate groups to provide insights into a potential link between siRNA production and the presence of high-impact variation. Density of high-impact variants was determined by calculating the proportion of sequence variants with frameshift or stop gained for each gene in each group. Our results show that the control group was absent from high-impact variants, and the non-siRNA-*PPR*s were also highly conserved with a median value of 0 (Fig. 4a). Compared with non-siRNA producing-*PPR*s, the density of high-impact variants was significantly higher in the hotspot siRNA-producing *PPR*s. Non-hotpot-siRNA-producing *PPR*s also had a higher median value of 0.00222, although not statistically different from non-siRNA *PPR*s (Fig. 4a). Both siRNA-producing and non-siRNA-producing *NLR* genes showed the highest density of high-impact variants. These results indicate a potential contribution of gene clustering to promoting diversification of siRNA-producing *PPR*s.

From the analysis of high-impact variations, we detected instances of pseudogenization in hotspot siRNA-producing *PPR* genes. Although these transcripts are inactivated for protein-coding ability, they continue to spawn siRNAs (Fig. 4b–e; Supplementary Fig. 6). One such instance involves homologs of the Col-0 gene *AT1G62670*, which has two homologous sequences in the accession Cvi-1. Both sequences are located within the *PPR*-siRNA hotspot region in Chromosome 1 of Cvi-1 and share similarities of 99.16% and 90.38% with Col-0 *AT1G62670*, respectively (Fig. 4b). One of the homologs, *ATCVI-1G76280*, is predicted to encode a PPR protein, while the other, named *ATCVI-1G76280-2*, represents a non-coding locus in an intergenic region of the plus strand. These two loci are situated 84,374 bp apart. *ATCVI-1G76280-2* appears to have experienced one AT deletion at the nucleotide position 501–502 of *ATCVI-1G76280*, resulting in a premature stop codon (Fig. 4b). Importantly, both Cvi-1 loci spawn siRNAs; however, due to sequence diversification, they produce distinct populations of siRNAs with specific sequences (Fig. 4c). Less than 10% of the siRNA populations was shared by both loci (Fig. 4d). Target prediction of these two loci revealed potential target sites of the same sRNA triggers (Supplementary Data 4). Importantly, abundant siRNAs were produced from similar regions between the target sites of TAS2 3′ D6 (–) and miR161.1 in both loci (Fig. 4e). Phasing patterns of the siRNAs are consistent with TAS2 3′ D6 (–) and miR161.1 being the major triggers for siRNA production in both loci (Supplementary Data 4), indicating a preservation of siRNA production mechanism despite gene duplication and pseudogenization.

A similar example was found in the accession Sha. In this case, two loci share sequence similarity with the Col-0 gene *AT1G63080* (Supplementary Fig. 6a). One of the homologous sequences has a premature stop codon due to a C-to-G point mutation at the nucleotide position 880 (Supplementary Fig. 6a). Both loci produce siRNAs, but their siRNA populations are highly specific due to sequence divergence (Supplementary Fig. 6b-c). Nevertheless, siRNA productions from these two loci are triggered by the same sRNAs with similar phasing patterns (Supplementary Fig. 6d; Supplementary Data 4). Taken together, these observations suggest that an accelerated diversification of *PPR*-siRNA-producing sequences could be achieved through sequence diversification, gene duplication, and high-impact variation including pseudogenization.

Given that *PPR* transcripts could potentially produce both proteins and siRNAs, we conducted experiments to clarify the role of siRNA production in the defense contribution of *PPR* genes. *PPR*-siRNAs have been previously reported to enhance *A. thaliana* defense against the oomycete pathogen *P. capsici*, likely by silencing target gene(s) in the invading pathogen^16^. In particular, siR1310 and siR1310-like *PPR*-siRNAs (collectively called siR1310 hereafter) are predicted to silence the *P. capsici* gene *Phyca_554980* and artificial introduction of siR1310 into *P. capsici* significantly reduced the virulence in *A. thaliana*^16^. Furthermore, an *A. thaliana* mutant of a siR1310-producing *PPR*, *AT1G62914*, exhibited increased susceptibility to *P. capsici*^16^. We generated complementation lines in the *at1g62914* mutant background by introducing a mutated sequence of *AT1G62914* (*62914 pm*), which contains a T-to-A point mutation at the nucleotide position 432 to implement a premature stop codon (Supplementary Fig. 7a**;** Supplementary Data 5). Although the full-length mRNAs could still be produced in the complementation lines, western blotting failed to detect protein products, likely due to the premature stop codon (Supplementary Fig. 7b-c). We confirmed that the production of siR1310 was restored in the *62914 pm* complementation lines to the wildtype level (Fig. 4f). Furthermore, the *62914 pm* construct also restored the hypersusceptibility phenotype of the *at1g62914* mutant to *P. capsici* (Fig. 4g). These results suggest that pseudogenized *PPR* loci could still contribute to plant defense, highlighting the functional significance of siRNA production of *PPR* genes in plant defense.

### siRNA-producing PPRs form an ancient clade that exhibits within-species diversification

PPR proteins are found in all eukaryotes with an expansion in plants^18^. Previously, siRNA production from *PPR* transcripts has been observed in 32 dicot plants^27,45,46^. We examined *PPR*-siRNA production in 55 plant species, including three early plants, ten monocots, and 42 eudicots (Supplementary Fig. 8a). These species were chosen based on the availability of high-quality reference genomes, gene annotations, and sRNA-seq datasets (Supplementary Data 6). To accommodate variation in genome annotations and updates in genome assemblies, PPR proteins from each genome were re-annotated based on sequence similarity using BLASTP against previously identified PPR proteins (https://ppr.plantenergy.uwa.edu.au/)^34^. In general, each species has at least one gene encoding each of the four PPR subfamily proteins, and up to 150 DYW class, 489 E class, 589 P class and 322 PLS class proteins (Supplementary Data 6).

To detect common themes in siRNA-producing *PPR*s in these evolutionarily diverse species, 21-nt siRNAs were mapped to a specific *PPR* locus in each species. A *PPR* locus was considered to produce siRNAs using a cutoff of TPM > 5. *PPR*-siRNAs were detected from all but five species. In most species where *PPR*-siRNAs were detectable, most of them are spawned from P class PPR-encoding genes (Supplementary Fig. 8b; Supplementary Data 6). These results indicate that *PPR*-siRNA production from P class PPR-encoding genes is an evolutionarily conserved phenomenon in plants. At this time, we cannot exclude the possibility that the lack of *PPR*-siRNA detection from the five species, including two dicots, two monocots and one early plant, could be due to limited resources in sRNA analysis.

**Fig. 5:**
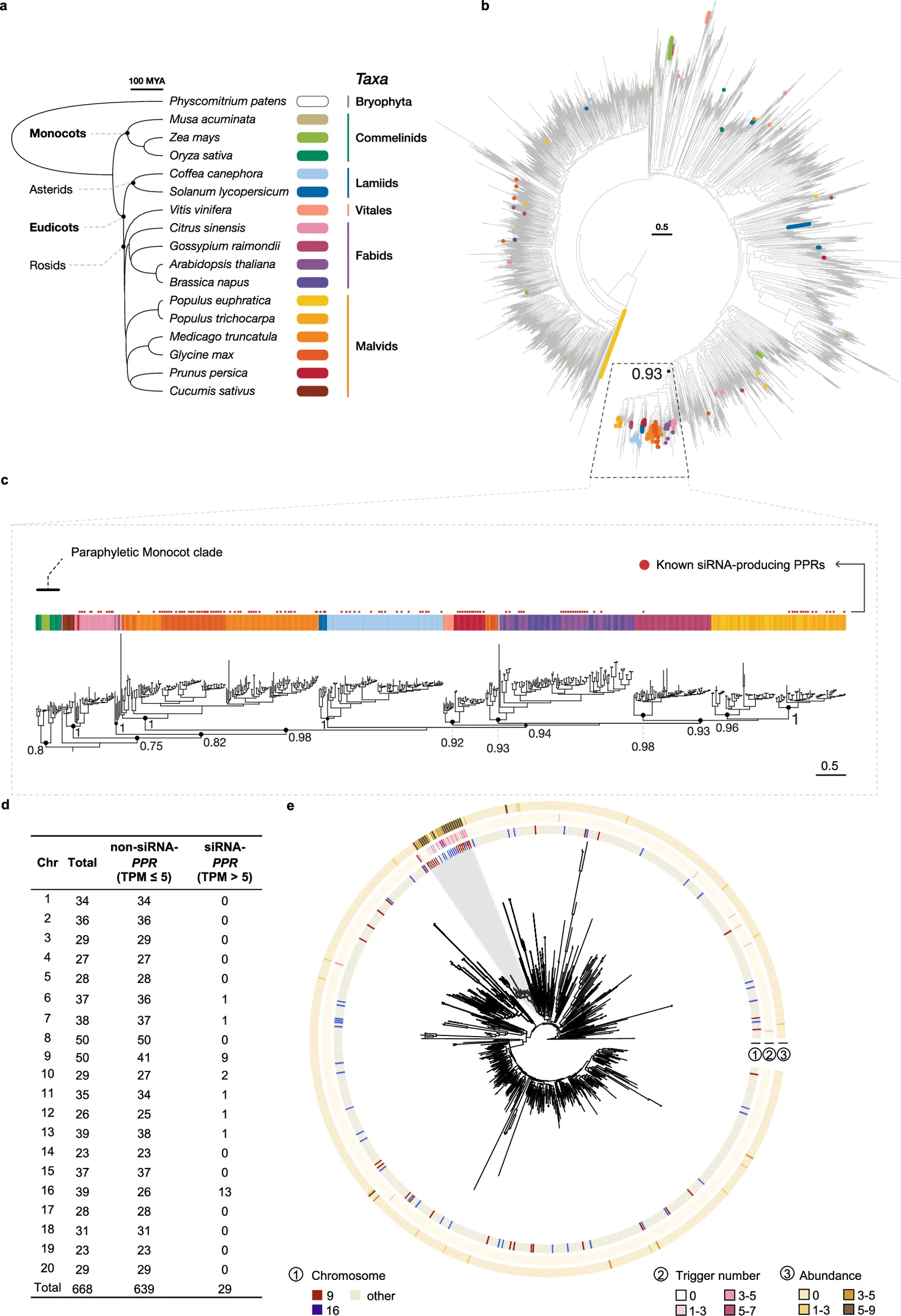
siRNA-producing *PPR*s form an evolutionarily conserved monophyletic clade. **a** Taxonomic tree of 17 representative species across land plants. **b** Phylogenetic tree of 11,617 PPRs from 17 plant species using amino acid sequences. A total of 168 sRNA-producing PPRs are highlighted by red dots. Four PPRs, including CCM1_CANAW from *Candida albicans*, CCM1_SCHPO from *Schizosaccharomyces pombe*, and LPPRC from both *Homo sapiens* and *Rattus norvegicus*, were used as the outgroups. **c** Phylogenetic tree of 546 PPRs belonging to the siRNA-producing clade with 122 known siRNA-producing PPRs highlighted by red dots. Annotation bars for each PPR are colored by the species of origin. Numbers next to the nodes shown on the phylogenetic trees indicate bootstrap value. **d** Number of siRNA-producing and non-siRNA-producing *PPR*s identified in each soybean Chromosome. **e** Phylogenetic tree of the entire complement of 668 soybean PPRs based on amino acid sequences. Ring 1 indicates the chromosomal locations of the genes. Ring 2 indicates the number of predicted miRNA target sites in each *PPR*. Ring 3 presents the log_2_-tranformed TPM of 21-nt siRNAs derived from each *PPR* based on sRNA-seq data from soybean leaves^86^. A Source Data file is also provided.

We further investigated the evolutionary relationship of the siRNA-producing PPRs. A phylogenetic tree of 11,617 PPR sequences from 17 representative species spanning major land plant lineages, including monocots and eudicots, was generated (Fig. 5a, b; Supplementary Data 7). We incorporated 168 PPRs that are evident to produce siRNAs from publicly available databases into this tree, which revealed that 122 of the siRNA-producing PPRs are clustered in a single clade (Fig. 5b; Supplementary Data 8). This remarkable result suggests that siRNA production is an ancient function of this specific PPR subfamily. We further examined the 546 PPRs from this “siRNA-producing” clade, color-coded by the species of origin (Fig. 5c). This analysis revealed a strong pattern of within-species diversification in this clade, which is consistent with their role in plant defense. Interestingly, we found a monocot sub-clade, with 22 PPRs from rice and maize that are not known to produce siRNAs, paraphyletic to the dicot clade containing siRNA-producing PPRs (Fig. 5c). Although siRNAs can be detected from monocot *PPR*s (Supplementary Fig. 8), these genes are scattered in the phylogenetic tree and do not belong to the “siRNA-producing clade”.

We next examined whether the siRNA-producing *PPR*s are also physically clustered in other species as observed in *A. thaliana*. For this purpose, soybean (Genome assembly, Glycine_max_v4.0) was analyzed. We predicted 668 PPR-encoding genes from the soybean genome, including 29 that produce 21-nt siRNAs (Fig. 5d; Supplementary Data 9). Twenty-three of the siRNA-producing genes putatively encode P class PPR proteins and belong to a single phylogenetic clade. Long branches in the phylogenetic tree of this clade indicate intra-species diversification of the genes and members in this phylogenetic clade are predicted to be prominent targets of sRNAs that can trigger siRNA production (Fig. 5e Ring 2). Furthermore, these genes are responsible for the production of 84.30% of the total 21-nt *PPR-*siRNA population in soybean (Fig. 5e Ring 3; Supplementary Data 9). All of these features are reminiscent to what we have observed from the siRNA-producing *PPR*s in *A. thaliana*.

Interestingly, not only the major soybean siRNA-producing *PPR*s form a single phylogenetic group, they are also physically linked as two clusters (Fig. 5d; Fig. 5e Ring 1; Supplementary Data 9): eight of the nine siRNA-producing *PPR*s on Chromosome 9 are located within a ~7 Mb region (nucleotides 40,537,248–47,957,179) and the 13 siRNA-producing *PPR*s on Chromosome 16 are all located within a ~6 Mb region (nucleotides 29,931,447–36,307,342). These results indicate that, like in *A. thaliana*, soybean also employs designated *PPR* gene clusters as hotspots for siRNA production.

### Heterologous expression of *PPR*-siRNAs in *N. benthamiana* enhances disease resistance

Considering *PPR*-siRNA production as an evolutionarily conserved process in plants, we explored how plant defense can be elevated by enhancing production of these antimicrobial agents. As a proof-of-concept, we designed a *PPR*-siRNA-producing cassette by co-expressing sRNA trigger(s) and a *PPR*-siRNA-producing sequence from *A. thaliana* in *N. benthamiana* (Supplementary Fig. 9a). To exclude potential involvement of protein product(s), *AT1G62914pm* was used as the siRNA producing sequence. Target prediction in *AT1G62914pm* using 158 known miRNAs produced by *N. benthamiana*^47^ did not retrieve any significant hits. Therefore, we co-expressed *AT1G62914pm* with sRNA triggers from *A. thaliana*, in particular miR161 and TAS2 3′ D6 (–), which are associated with the production of some of the highest siRNA-producing *PPR*s^29^. As a control, we generated a variant of *AT1G62914*, referred to as *AT1G62914del*, which contains a deletion of a 159 bp (631 to 789 nt) fragment from which most siRNAs, including siR1310, are produced in wildtype *AT1G62914* (Supplementary Fig. 7a; Supplementary Fig. 9a; Supplementary Data 5). Importantly this mutant gene still retains the target sites of the sRNA triggers.

**Fig. 6:**
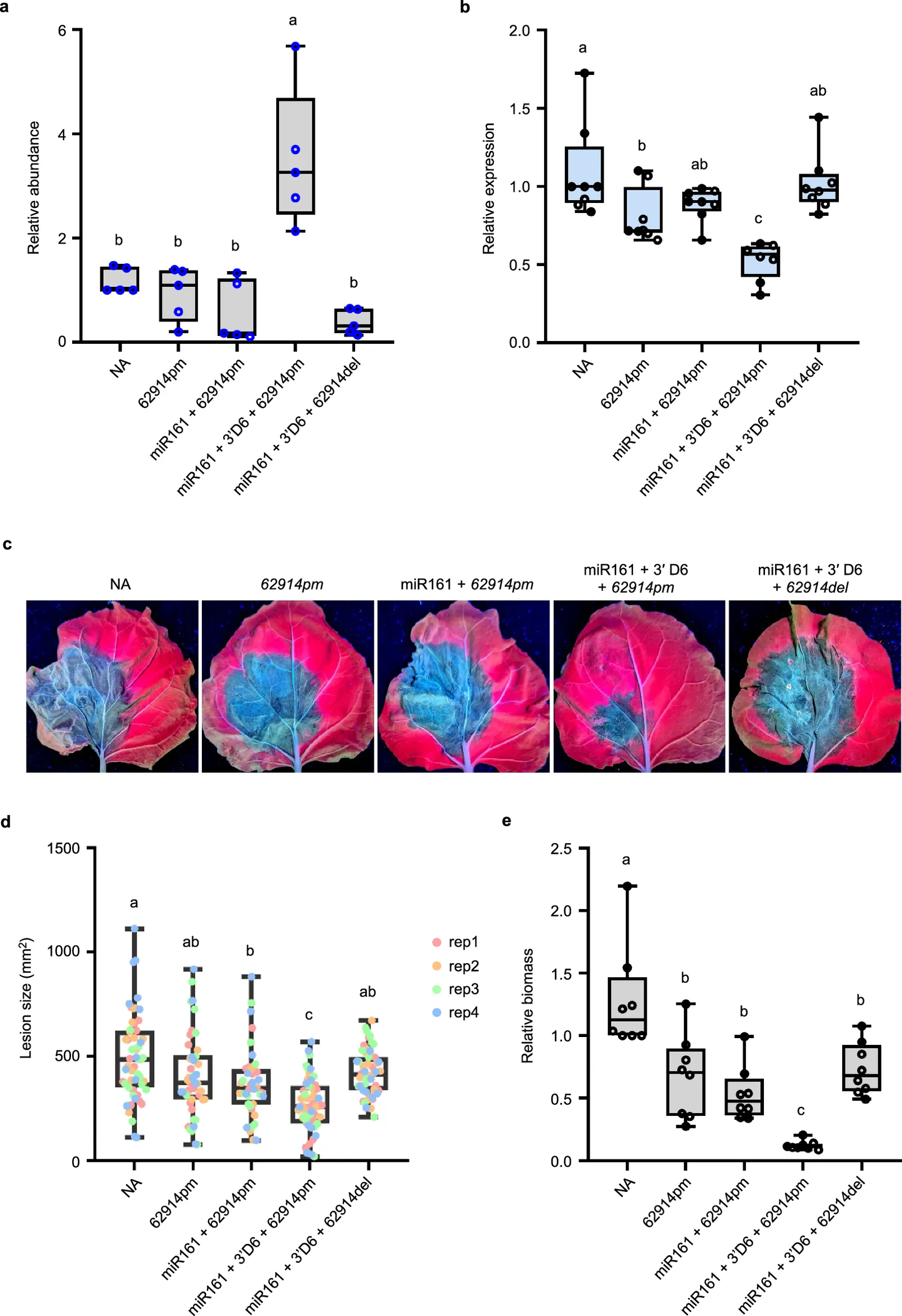
Ectopic expression of a *PPR*-siRNA-producing cassette in *N. benthamiana* enabled siRNA accumulation and enhanced resistance to *P. capsici.* **a** Relative abundance of siR1310 in *N. benthamiana* leaves determined by stem loop qRT-PCR. Different combinations of *Agrobacterium* carrying *AT1G62914pm* or *AT1G62914del* with the sRNA triggers miR161 or TAS2 3′ D6 (–) were infiltrated into *N. benthamiana* leaves, which were subsequently inoculated with *P. capsici* zoospores at 24 h after Agro-infiltration. Leaf tissues were collected at 3 days post *P. capsici* infection (dpi) for further analyses. NA, no Agro-infiltration; *62914del*, *AT1G62914* with a 159 bp deletion (631–789 nt) that eliminates siR1310 production without affecting the target sites of sRNA triggers. Data are presented as mean ± SD (*n* = 5 independent biological replicates). **b** Transcript abundances of the *P. capsici* gene *Phyca_554980* determined by qRT-PCR. Data are presented as mean ± SD (*n* = 8 independent biological replicates). **c** Disease symptoms on *N. benthamiana* leaves at 3 dpi. **d** Lesion sizes on the inoculated *N. benthamiana* leaves measured at 3 dpi. At least ten leaves per treatment were examined across four independent biologial replicates. Data are presented as mean ± SD. **e** Biomass of *P. capsici* in inoculated *N. benthamiana* leaves determined at 3 dpi. Data are presented as mean ± SD (*n* = 8 independent biological replicates). For all experiments in (**a**, **b**, **d**, **e**), statistically significant differences were determined using one-way ANOVA with Tukey’s post hoc test and labeled by different letters (*P* < 0.05). Adjusted *P* values and test statistics are provided in the Source Data file.

When *AT1G62914pm* was co-expressed with both miR161 and TAS2 3′ D6 (–), but not miR161 alone, we detected an increased accumulation of *PPR*-siRNAs in *N. benthamiana*, as indicated by an elevated level of siR1310 (Fig. 6a). In contrast, co-expression of the trigger sRNAs with *AT1G62914del* did not increase siR1310 accumulation. We then inoculated the *N. benthamiana* leaves using zoospore suspension of *P. capsici*. siR1310 was previously shown to silence the *P. capsici* gene *Phyca_554980*^16^. Consistent with the increased accumulation of siR1310 in tissue co-expressing *AT1G62914pm*, miR161 and TAS2 3′ D6 (–), we observed reduced transcript levels of *Phyca_554980* (Fig. 6b) and enhanced resistance to *P. capsici*, reflected by reduced lesion sizes (Fig. 6c, d) and pathogen biomass (Fig. 6e).

To further support the specificity of defense based on *AT1G62914pm*-derived siRNAs, we inoculated *N. benthamiana* expressing the same siRNA-producing cassette by *Phytophthora infestans*. Target prediction indicates that the *Phyca_554980* homolog in *P. infestans* is divergent in sequence and cannot be silenced by *PPR*-derived siRNAs, including siR1310 and siR0513, that are known to silence *Phyca_554980* (Supplementary Fig. 9b). Consistent with this prediction, co-expression of *AT1G62914pm* with miR161 and TAS2 3′ D6 (–) did not reduce lesion size (Supplementary Fig. 9c-9d), indicating that this defense requires specific siRNA-target interactions. Together, these results demonstrate that engineering *PPR*-siRNAs can be employed as a promising immune enhancement strategy.

## Discussion

The secondary siRNA pathway has emerged as a major contributor to plant defense. This defense function could be attributed to two different mechanisms: (i) siRNAs produced from *NLR* and *TAS3* transcripts might regulate the expression of their endogenous targets to fine-tune immune-related gene expression^10–15^; (ii) siRNAs produced from *PPR* and *TAS1/TAS2* transcripts may silence target genes in invading eukaryotic pathogens through trans-species gene silencing^16^. Unlike endogenous gene targets, pathogen transcripts targeted by plant sRNAs are presumed to be under selective pressure to evade this defense^28^. Therefore, source sequences that produce siRNAs directly targeting pathogen genes may also undergo accelerated evolution, thus exhibiting different evolutionary trajectories from those producing siRNAs regulating endogenous targets. In this study, we show that siRNA-source *PPR*s form an evolutionarily conserved clade that exhibits remarkable within-species diversification, which is in stark contrast to the highly conserved non-siRNA-source *PPR* genes. This difference is consistent with a distinct selection regime imposed by host-pathogen arms race to siRNA-source *PPR*s.

*PPR*s represent one of the largest gene families in land plants^18^. The best studied function of PPRs is to regulate mitochondrial genes responsible for cytoplasmic male sterility (CMS) as RNA-binding proteins^19,21^. In *A. thaliana*, most siRNA-producing *PPR* genes belong to the “Restorer of Fertility-Like” (RFL) group. However, not all RFLs are siRNA-producers: in Col-0, 16 of the 20 siRNA-producing *PPR*s were classified as *RFL*s, and 16 out of 26 *RFL*s produce siRNAs^25^. Notably, five *RFL* genes located within the siRNA-producing hotspot on Chromosome 1 have been shown to possess RNA-binding activity^48^. Furthermore, the loss of *RFL8* (*AT1G62720*) resulted in an embryo-lethal phenotype, consistent with a role in regulating CMS^49^, however, *RFL8* does not produce siRNAs. In contrast, knockout mutants of the other four *RFL* genes, all of which produce siRNAs, did not exhibit developmental defects. Therefore, while these genes may still contribute to CMS regulation, their ability to produce siRNAs is not readily explained by this function alone.

RFLs were previously reported to exhibit intraspecific diversification, which was attributed to nuclear-cytoplasmic conflict^25,26^. *RFL* genes are widely produced by flowering plants and the diversification was observed in both monocots and dicots. In contrast, although rice and maize encode *PPR*s belonging to the siRNA-producing clade, they are not known to produce siRNAs. Intriguingly, our analysis indicates that within-species diversification of siRNA-producing *PPR*s is only apparent in dicots. This distinctive feature between *RFL*s and siRNA-producing *PPR*s suggests that the expansion and diversification of *PPR* genes may not only associate with fertility restoration but also siRNA-mediated defense. An involvement of PPRs in plant defense is also supported by transcriptomic studies in which multiple PPRs were up-regulated after immune activation in poplar^50^ or correlated with stronger immunity in lettuce after fungal infection^51^.

It has been hypothesized that secondary siRNA production is particularly effective in globally impacting genes belonging to large gene families by a small number of miRNAs through siRNA production from primarily targeted transcripts^10–14^. However, a previous transcriptomic analysis in *A. thaliana* did not find evidence that can support a major role of *PPR*-siRNAs in regulating their parental or other *PPR* gene expression^16^. In addition, siRNAs do not have a known function in regulating *RF* gene expression. We therefore propose a functional divergence of siRNA-producing and non-siRNA-producing *RFL* genes, with the former having evolved a role in immunity. The siRNA production function might have primarily evolved to maintain expression homeostasis among *PPR* genes, similar to what have been observed in *NLR* gene regulation. These siRNAs may have subsequently been repurposed to target pathogen genes, and this function would further impose a positive selection on the siRNA-producing *PPR*s reported in this study.

In both *A. thaliana* and soybean, the majority of siRNA-producing *PPR* genes are physically clustered, presumably promoting sequence diversification through repeated local duplication and rearrangement. This organization parallels that of *NLR* genes, which frequently form multi-gene clusters that are believed to facilitate the rapid expansion and diversification of pathogen recognition capacity^52–54^. A similar mechanism may promote the diversification of siRNA-producing sequences and the diversity of the siRNA repertoires they generate. In *A. thaliana*, the majority of the siRNA-producing *PPR*s located within a cluster on Chromosome 1 are highly similar ( > 78% identity) to a single gene (*AT1G63320*). These features are compatible with a scenario in which an *AT1G63320*-like ancestor seeded the expansion of this gene cluster. The subsequent diversification among its derivatives, while retaining the ability to bind the trigger sRNAs and generate siRNAs, contributes to the observed diversity in siRNA-producing sequences.

*PPR* genes can function either through their encoded protein products or siRNAs derived from transcripts. We found multiple pseudogenization events within the siRNA-producing *PPR* cluster across different *A. thaliana* accessions. Although these pseudogenized loci no longer encode intact proteins, they preserve the trigger sRNA target sites and maintain the ability to produce secondary siRNAs. Functional analysis confirmed that at least one pseudogenized *PPR* still confers disease resistance, indicating that its primary biological function is through anti-microbial siRNA production. Notably, pseudogenized *PPR*s produce siRNA populations that are substantially diverged from those produced by their intact homologs, indicating high-impact variations such as pseudogenization contribute to the expansion of the siRNA diversity, which is likely advantageous for the plants to defend against various pathogens in the surrounding environment^28,55^.

In *A. thaliana*, the secondary siRNA pathway has been implicated in defense against not only the oomycete pathogen *P. capsici*, but also the fungal pathogens *Botrytis cinerea* and *Verticillum dahliae*^2,56^, indicating a role in broad-spectrum disease resistance. As a counter-defense mechanism, both oomycete and fungal pathogens deploy effector proteins that suppress secondary siRNA accumulation^16,57^. In particular, the *Phytophthora* effector PSR2 suppresses the biogenesis of secondary siRNAs derived from *TAS1*, *TAS2*, and *PPR* transcripts in *A. thaliana*^16^, consistent with our observation that these siRNAs contribute to anti-*Phytophthora* defense through trans-species RNAi. A systematic analysis of siRNA target sites in these pathogens to determine whether they exhibit sequence diversification will provide insights into their evolutionary dynamics, which would lend further support to a host-pathogen arms race centered on siRNA-based defense strategy.

Our finding that the siRNA-producing PPRs belong to an evolutionarily conserved clade not only supports siRNA production as an ancient function of these PPRs, but also highlights the promise of engineering siRNA-producing sequences in a wide range of plants to enhance disease resistance. As a proof-of-concept, we introduced an *A. thaliana PPR*-siRNA-producing cassette into *N. benthamiana*. Expressing this cassette led to a significant accumulation of specific *PPR*-siRNAs and elevated resistance to *P. capsici*. Importantly, efficient siRNA production required co-expression of the siRNA-producing sequence with two sRNA triggers whose cleavage sites are aligned in the same 21-nt phase. In contrast, the well-known trigger sRNA miR161, by itself, was insufficient to initiate *PPR*-siRNA production. This is consistent with the observation that almost all siRNA-producing *PPR*s harbor predicted target sites for multiple distinct trigger sRNAs. It will be interesting to further optimize siRNA production by systematically exploring different combinations of trigger sRNAs with *PPR* genes. Equally interesting is to express trigger sRNAs with engineered siRNA-producing sequences that are designed to target selected genes in specific pathogens to test the efficacy of disease resistance.

In summary, this study identified a specific *PPR* gene cluster as a conserved source of siRNAs that confer trans-species silencing of pathogen genes. This discovery highlights an exciting opportunity to develop disease resistant crops through bespoke production of anti-microbial sRNAs by engineering the siRNA-producing *PPR* sequences. Future investigations of sRNA profiling from a wider range of plant species, especially in monocots and early green lineages, as well as in different tissues/organs and under various biotic stress conditions, will help establish a deeper understanding of *PPR*-siRNAs as an integral component of plant immune system. A broader role of *PPR*-siRNAs in conferring broad-spectrum resistance should also be examined to understand how widely this natural defense mechanism can be deployed to enhance disease management in economically important crops. Finally, potential fine-tuning of *RF* gene expression by *PPR*-siRNAs should be carefully evaluated to clarify the multi-functionality of PPRs in regulating both reproductive development and defense.

## Methods

### Reference genome and gene annotation sources

The genome sequence (TAIR10) and gene annotation (Araport11) of *A. thaliana* Col-0 accession were obtained from EnsemblPlant (release 56)^58^. The Ct-1 genome was sequenced using Illumina paired-end reads, and both its genome sequence and gene annotation were derived from the 19 genomes project^31^. The genome sequences of An-1, Cvi-1, Eri-1, Kyo-1, L*er*-0, and Sha were generated through high resolution PacBio sequencing^32^ and their annotations were obtained from the 1001 Genomes Project^59^. The genome sequences and gene annotations for 54 additional plant species were retrieved from public databases, including NCBI, EnsemblPlant (release 56), and Phytozome v13^60^ (Supplementary Data 6).

For *A. thaliana* Col-0, annotations of eight *TAS* loci (*TAS1a/b/c*, *TAS2*, *TAS3a/b/c* and *TAS4*) and 27,655 protein-coding genes were obtained from EnsemblPlant (release 56)^58^. Amino acid sequences of 475 PPRs were downloaded from the “PPR” website^34^. A total of 167 NLRs were annotated using the nlrsnake software (https://github.com/TeamMacLean/nlrsnake)^61^. Homologs of *TAS* loci, *PPR*s, and *NLR*s in *A. thaliana* Col-0 in the other seven accessions were identified as described in the “Identification of homologous genes” section.

### sRNA-seq data analysis

Rosette leaves from 2-week-old *A. thaliana* plants were used for sRNA library construction. Libraries were prepared from total RNAs using the NEBNext Multiplex Small RNA Library Prep Set for Illumina (E7300S) and sequenced on an Illumina Hiseq 4000 platform to generate 150 bp single-end reads. Before analysis, the raw sequencing reads in FASTQ format were removed the adapter sequence AGATCGGAAGAG using the software Cutadapt v3.2^62^. Any reads not successfully trimmed or containing more than one ‘N’ base were discarded by the adapter removal process. Quality filtering was performed by Trimmomatic v0.36^63^ using single-end mode with LEADING:3, TRAILING:3, SLIDINGWINDOW:4:15, MINLEN:18, and MAXLEN:28. After quality control, the resulting reads with lengths ranging from 18- to 28-nt were initially mapped to known structural RNA sequences from Rfam v14.9^64^, including transfer RNAs (tRNAs), ribosomal RNAs (rRNAs), small nuclear RNAs (snRNAs), and small nucleolar RNAs (snoRNAs). The remaining unmapped reads were secondly mapped to the *A. thaliana* reference genome using Bowtie v4.4.6^65^, allowing all alignments (-a) and zero mismatch (-v 0) per read. The successfully mapped reads represent clean reads.

For each *A. thaliana* accession, the diversity of siRNA population spawned from a given locus was assessed as the proportion of unique 21-nt siRNAs produced from that gene relative to the total number of unique siRNAs in that accession. sRNA-seq data from other plant species were processed and analyzed using the same parameters and pipeline as described above for *A. thaliana*. For each locus, sRNA abundance (TPM) was normalized by dividing the number of sRNAs mapped to that gene by the total number of genome-matched clean reads in the library.

### Identification of homologous genes

Identification of homologs of Col-0 protein-coding genes in other *A. thaliana* accessions was conducted using SimpleSynteny (v1.3.2)^35^, BLASTN and BLASTP (BLAST+ , v2.9.0)^36^ using default parameters. In total, 27,377 Col-0 genes, excluding mitochondrial and chloroplast genes and CDS sequences shorter than 80 aa, were used as queries. Initial homolog candidates were retained if they met all of the following criteria: query coverage > 30%, subject coverage > 30%, identity > 60%, and bit score > 50. The remaining homologs were filtered based on the scoring system Equation ( 1),

1 $${{{\rm{Score}}}}=\frac{{{{{\rm{Coverage}}}}}_{{{{\rm{query}}}}}\times {{{{\rm{Coverage}}}}}_{{{{\rm{subject}}}}}\times {{{{\rm{Score}}}}}_{{{{\rm{bit}}}}}}{{{{{\rm{Gap}}}}}_{{{{\rm{num}}}}}+\,1}$$

In this formula, ${{{{\rm{Coverage}}}}}_{{{{\rm{query}}}}}$ and ${{{\rm{C}}}}{{{{\rm{overage}}}}}_{{{{\rm{subject}}}}}$ indicate the coverage of the aligned query and subject sequence, respectively. ${{{\rm{Scor}}}}{{{{\rm{e}}}}}_{{{{\rm{bit}}}}}$ reflects the overall quality of the alignment. ${{{{\rm{Gap}}}}}_{{{{\rm{num}}}}}$ represents the number of gaps in the alignment. Higher scores indicate more reliable homologies.

By retaining the highest score hit for each Col-0 gene, 26,877, 26,831, 26,753, 26,892, 26,887, 26,874, and 26,828 homologs for the 27,377 Col-0 genes were identified in An-1, Ct-1, Cvi-1, Eri-1, Kyo-1, L*er*-0, and Sha, respectively. Among these, 467, 471, 459, 465, 464, 467, and 461 genes were homologous to the 475 *PPR*s in Col-0, while 140, 144, 122, 140, 129, 137, and 133 genes were homologous to the 167 *NLR*s in Col-0.

Homologs of non-coding genes in Col-0, including eight *TAS* loci (*TAS1a/b/c, TAS2, TAS3a/b/c*, and *TAS4*) and 11 sRNA triggers, were identified in the other seven *A. thaliana* accessions as the best hits with the highest sequence similarity. These 11 sRNA triggers including miR161.1, miR161.2, miR400, TAS1a 3′ D9 (–), TAS1b 3′ D4 (–), TAS1c 3′ D6 (–), TAS1c 3′ D10 (–), TAS2 3′ D6 (–), TAS2 3′ D9 (–), TAS2 3′ D11 (–), and TAS2 3′ D12 (–) were obtained from both miRBase^66^ and a previous study^42^.

Sequence logos were generated for the 11 trigger sRNAs using homologous sequences identified in eight *A. thaliana* accessions. sRNA sequences with the highest sequence similarity with the Col-0 homolog were retrieved using genome alignments. Their accumulation in each accession was validated using sRNA sequencing reads. All sRNAs, except for TAS1b 3′ D4 (–) in Ct-1, were supported by high-quality reads. Almost all sRNAs exhibit consistent numbers of mapped loci across the eight accessions. The only exception is TAS1c 3′ D10 (–), which has three mapped loci in Cvi-1, one mapped locus in An-1, and two mapped loci in the other six accessions. The read counts and the number of mapped loci for all the sRNA triggers are provided in the “sRNA Triggers” sheet of Supplementary Data 4.

### Determination of sRNA targeting scores on *PPR*s

The GSTAr.pl algorithm (version 1.0)^47^ and psRNATarget^67^ were utilized to predict targeting relationships for sRNAs on *PPR* transcripts in all eight *A. thaliana* accessions and in soybean. Genes with an AllenScore < 4 or an Expectation score ≤ 5 were defined as predicted targets.

### Nucleotide diversity analysis

Nucleotide diversity was analyzed for six gene groups across eight *A. thaliana* accessions. The hotspot-siRNA-*PPR*, non-hotspot-siRNA-*PPR*, non-siRNA-*PPR*, siRNA-*NLR*, non-siRNA-*NLR* groups included 14, 4, 457, 4, and 163 genes, respectively, as well as a control group consisting of 500 randomly selected genes. The control group was generated by performing 1000 random samplings, each containing 500 genes. Nucleotide diversity was evaluated by determining Pi, Watterson’s theta, and Distance values.

For each gene, codon-based alignment (https://github.com/lyy005/codon_alignment) was conducted on the CDS sequences of homologs from the eight *A. thaliana* accessions. If one or more accessions lacked a specific ortholog, those accessions were excluded from the calculation for that gene to avoid skewing in assessing the diversity. Pi and Watterson’s theta were estimated using the sliding.window.transform() in the R package PopGenome^68^ with a sliding window size of 50 and a step size of 50. The Distance value was calculated using the dist.dna function in the R package ape^69^.

Correlation between the sequence diversity in *PPR*, *NLR*, or *TAS* genes and the diversity of siRNAs spawned from the corresponding loci was evaluated using the cor.test function in the R package stats^70^. The diversity of siRNAs spawned from each gene in the eight *A. thaliana* accessions was calculated as the proportion of unique 21-nt siRNAs produced from that gene relative to the total number of unique 21-nt siRNAs in that accession. Spearman’s rank correlation was used to evaluate the strength and direction of associations between variables and *P* < 0.05 was considered statistically significant.

### PPR identification and Phylogenetic analyses

Phylogenetic trees for PPRs in *A. thaliana* and soybean were constructed using the amino acid sequences of 475 PPRs from the Col-0 accession and 668 PPRs from soybean, respectively. PPR sequences were aligned using MUSCLE (v3.8.31)^71^ and automatically trimmed with trimAl (v1.2rev59)^72^. Phylogenetic trees were inferred with IQ-TREE2 (v2.0.4)^73^ using the VT+F+G4 model for *A. thaliana* and the LG+F+G4 model for soybean, with 1,000 ultrafast bootstrap replicates. Trees were visualized using the R package ggtree (v3.6.2)^74^.

The species phylogeny was constructed from the amino acid sequences of AT2G21710 orthologs, which encode a mitochondrial transcription termination factor. The phylogenetic relationships among the 55 plant species are consistent with a previous report^75^. This tree was inferred with IQ-TREE2 using the JTTDCMut+I+G4 model.

Pentatricopeptide Repeat (PPR) proteins were identified by searching four Pfam HMM profiles (PF01535, PF12854, PF13041, PF13812) against 17 RefSeq proteomes (Supplementary Data 7) using hmmsearch --max^76^. A total of 19,352 sequences with hits to at least one of these profiles and an E-value ≤ 0.05 were retained. To ensure data quality, sequences shorter than 150 amino acids or longer than 1500 amino acids were excluded, leaving 19,274 sequences for further analysis. Redundant sequences were removed using the “unique” function of the BiocGenerics R package^77^, resulting in a final dataset of 11,617 unique PPR sequences (Supplementary Data 8).

For phylogenetic analysis, we incorporated 168 reference sRNA-producing PPRs and four outgroup PPRs, including CCM1_CANAW from *Candida albicans*, CCM1_SCHPO from *Schizosaccharomyces pombe*, and LPPRC from both *Homo sapiens* and *Rattus norvegicus* into the extracted PPR sequences (Supplementary Data 8). These sequences were aligned using FAMSA (v2.2.2) with the default options^78^, and the phylogenetic tree was constructed with FastTree (v2.1.10) using the LG model^79^. This phylogenetic tree was rooted at the LPPRC sequences. The sRNA-producing clade was identified based on a well-supported branch within this phylogeny. Among the 546 sequences in this clade, 22 are from a paraphyletic clade with two monocot plant species, and 122 are known siRNA-producing PPRs. These sequences were realigned using MAFFT (v7.525)^80^ and a new phylogenetic tree was constructed using FastTree (v2.1.11)^79^ and rooted at the paraphyletic clade. All the phylogenetic trees were visualized and manually curated using iTOL^81^. The taxonomic tree for the 17 selected species was retrieved from TimeTree 5 (timetree.org)^82^.

### High-impact variant density analysis

Whole genome sequence variation data in 1,135 *A. thaliana* accessions were downloaded from the 1001 Genomes Project^83^. High-impact variants were characterized by their potential to trigger deleterious effects on gene functions, such as introducing premature stop codons through single nucleotide polymorphisms (SNPs) or causing frameshift mutations through small insertions or deletions.

High-impact effects were assessed in six gene groups: hotspot-siRNA-*PPR*s, non-hotspot-siRNA-*PPR*s, non-siRNA-*PPRs*, siRNA-*NLR*s, non-siRNA-*NLR*s, and random genes. For each gene, the high-impact variant density was determined by the proportion of variants carrying frameshift and stop codon gained within its coding sequence.

### Plant growth and *Phytophthora* infection

Seeds for this study were ordered from the Arabidopsis Biological Resource Center (ABRC) and included CS97808 (An-1), CS76786 (Ct-1), CS97810 (Cvi-1), CS97813 (Eri-1), CS97807 (Kyo-1), CS97814 (L*er*-0), CS97811 (Sha), and CS737432 (*62914mut*). Plants were grown in a growth room with controlled environment at 22 ± 2 °C and 12 h light/12 h dark photoperiod.

*Phytophthora capsici* isolate LT263 was cultured and used for plant inoculation by zoospore suspensions as previously described^16^. Briefly, *P. capsici* was grown on 10% V8 agar at room temperature (RT) in the dark for 3–4 days. Mycelial plugs were then transferred to 10% V8 broth and incubated at RT in the dark for 2 days. The plugs were washed three times with sterile water, then kept in sterile water at RT in the dark for 24 h. Subsequently, they were exposed to light at RT for 24 h, followed by a cold shock (4 °C, 30 min) and then illumination at RT for 30 min to trigger zoospore release. The suspension was filtered through one layer of Miracloth to remove agar and mycelial debris. The final zoospore concentration was adjusted to 10^6^ zoospores/mL for inoculation. Biomass of *P. capsici* was determined by qPCR at 3 days post inoculation. Primers used for the PCR analysis are listed in Supplementary Data 10.

### Construction of *AT1G62914pm* and *AT1G62914del*

To construct expression lines of *AT1G62914* carrying a premature stop codon, the coding sequences of *AT1G62914* from *A. thaliana* Col-0 were amplified using gene-specific primers (Supplementary Data 10). The PCR products were cloned into the pENTR/D-TOPO vector and site-directed mutagenesis was performed to generate *AT1G62914pm*, which was cloned into the vector pEG104 using Gateway cloning (Invitrogen). To generate the *AT1G62914del* construct, two fragments of *AT1G62914* flanking the deleted region were amplified and fused at the *Bsa*I sites and the resulting fragments were cloned into pEG104 using In-Fusion cloning. Primers used to amplify the trigger sRNAs for cloning were also included in Supplementary Data 10.

### Stem-loop qRT-PCR

The abundance of siR1310 in *A. thaliana* or *N. benthamiana* was quantified by stem-loop qRT-PCR using 1.0 μg of total RNA from leaves as described previously^84^. Briefly, during reverse transcription, the primer anneals specifically to the target small RNA and is extended by reverse transcriptase, generating a longer cDNA that includes the stem-loop sequence. In the qPCR step, a forward primer specific to the mature small RNA and a universal reverse primer matching the stem-loop region are used for amplification. qRT-PCR was performed using SYBR Green PCR Master Mix (Thermo Scientific) and U6 as an internal control (Supplementary Data 10). For each biological replicate, the *Ct* (cycle threshold) was defined as the PCR cycle at which the fluorescence signal exceeded the detection threshold. Δ*Ct* was calculated by subtracting the mean *Ct* of the U6 transcripts as an internal control from the mean *Ct* of the sample (Δ*Ct* = mean *Ct*_sample_ − mean *Ct*_U6_). One replicate was designated as the reference (ΔΔ*Ct* = 0), and ΔΔ*Ct* for the remaining replicates was calculated relative to this reference (ΔΔ*Ct* = Δ*Ct*_replicate_ − Δ*Ct*_reference_). Relative abundance was calculated using the 2^-ΔΔ*Ct*^ method.

### Statistical analyses

All statistical analyses in this study were performed using JMP Pro (v13.0, SAS) or R software. Quantitative data are presented as mean ± standard deviation (SD). For comparisons among multiple experimental groups, data normality and homogeneity of variance were assessed prior to statistical testing. One-way analysis of variance (ANOVA) followed by Tukey’s honestly significant difference (HSD) post hoc test was performed for multiple pairwise comparisons in Fig. 4f, g, Fig. 6a, b, d, e, Supplementary Fig. 7b, and Supplementary Fig. 9d. Statistically significant differences (*P* < 0.05) are indicated by different letters. For non-parametric multi-group comparisons, the Kruskal-Wallis test with Bonferroni-adjusted post hoc comparisons was applied in Fig. 3a, c, Fig. 4a, and Supplementary Fig. 5a. Statistically significant differences are denoted by different letters based on adjusted *P* values (adjusted *P* < 0.05). Exact *P* values, degrees of freedom, and sample sizes (*n*, representing independent biological replicates or individual genes/leaves as specified in figure legends) for all statistical tests are provided in the Source Data file.

### Reporting summary

Further information on research design is available in the Nature Portfolio Reporting Summary linked to this article.

## Supplementary information

Supplementary Information

Description of additional supplementary files

Supplementary Data 1

Supplementary Data 2

Supplementary Data 3

Supplementary Data 4

Supplementary Data 5

Supplementary Data 6

Supplementary Data 7

Supplementary Data 8

Supplementary Data 9

Supplementary Data 10

Reporting Summary

Transparent Peer Review file

## Source data

Source Data

## Data Availability

The raw sRNA-seq data of *A. thaliana* accession Col-0 are available in the NCBI SRA under accession code SRP135923. Raw sRNA-seq data for the other seven *A. thaliana* accessions have been deposited in NCBI BioProject database under accession code PRJNA1037763. Source data are provided with this paper.

## Code availability

All custom R scripts and Jupyter Notebooks used for bioinformatics analyses and statistical evaluations in this study are available in the GitHub repository [https://github.com/fionaliii/PPR-siRNA] and have been archived in Zenodo under [https://doi.org/10.5281/zenodo.21687977]^85^.
