# Supplementary material for: A conserved small RNA-generating gene cluster undergoes sequence diversification and contributes to plant immunity
Source: Nat Commun. 2026 Aug 26;17:10187. doi: 10.1038/s41467-026-77164-1 (PMC13612597; doi:10.1038/s41467-026-77164-1)
Supplement: Supplementary file 1 [file 41467_2026_77164_MOESM13_ESM.pdf]

nature portfolio

Corresponding author(s): Wenbo Ma and Yingnan Hou

Last updated by author(s): Feb 9, 2026

Reporting Summary

Nature Portfolio wishes to improve the reproducibility of the work that we publish. This form provides structure for consistency and transparency in reporting. For further information on Nature Portfolio policies, see our Editorial Policies and the Editorial Policy Checklist.

Statistics

For all statistical analyses, confirm that the following items are present in the figure legend, table legend, main text, or Methods section.

n/a	Confirmed	
☐	☒	The exact sample size (*n*) for each experimental group/condition, given as a discrete number and unit of measurement
☐	☒	A statement on whether measurements were taken from distinct samples or whether the same sample was measured repeatedly
☐	☒	The statistical test(s) used AND whether they are one- or two-sided<br>*Only common tests should be described solely by name; describe more complex techniques in the Methods section.*
☒	☐	A description of all covariates tested
☐	☒	A description of any assumptions or corrections, such as tests of normality and adjustment for multiple comparisons
☐	☒	A full description of the statistical parameters including central tendency (e.g. means) or other basic estimates (e.g. regression coefficient) AND variation (e.g. standard deviation) or associated estimates of uncertainty (e.g. confidence intervals)
☐	☒	For null hypothesis testing, the test statistic (e.g. *F*, *t*, *r*) with confidence intervals, effect sizes, degrees of freedom and *P* value noted<br>*Give P values as exact values whenever suitable.*
☒	☐	For Bayesian analysis, information on the choice of priors and Markov chain Monte Carlo settings
☒	☐	For hierarchical and complex designs, identification of the appropriate level for tests and full reporting of outcomes
☐	☒	Estimates of effect sizes (e.g. Cohen's *d*, Pearson's *r*), indicating how they were calculated

Our web collection on statistics for biologists contains articles on many of the points above.

Software and code

Policy information about availability of computer code

Data collection

Plant Materials: Seeds of Arabidopsis thaliana accessions were obtained from the Arabidopsis Biological Resource Center (ABRC). Plants were grown under controlled environmental conditions (22 ± 2°C, 12h light/12h dark) without the use of software.

Genome and Annotation Data: Reference genome sequences and gene annotations were downloaded from public databases including EnsemblPlant v56, NCBI, Phytozome v13, and the 1001 Genomes Project. No specific software was used for data retrieval.

sRNA Library Preparation and Sequencing: Small RNA libraries were constructed using the NEBNext multiplex small RNA library prep set for Illumina (E7300S) following manufacturer protocols. Sequencing was performed on an Illumina HiSeq 4000 platform. No computational software was involved at this step.

Whole-Genome Variation Data: Genomic variation data for 1,135 Arabidopsis accessions were downloaded from the 1001 Genomes Project repository.

Data analysis

All custom R scripts and Jupyter Notebooks used for bioinformatics analyses and statistical evaluations in this study are available in the GitHub repository [https://github.com/fionaliii/PPR-siRNA] and have been archived in Zenodo under DOI [https://doi.org/10.5281/zenodo.21687977].

Cutadapt v3.2 for adapter trimming of raw sRNA-seq reads.

Trimmomatic v0.36 for quality filtering of sRNA-seq reads (single-end mode, parameters: LEADING:3, TRAILING:3, SLIDINGWINDOW:4:15, MINLEN:18, MAXLEN:28).

Bowtie v4.4.6 for mapping cleaned sRNA reads to the reference genome (parameters: all alignments allowed, 0 mismatches).

SimpleSynteny v1.3.2 for identification of homologous genes between A. thaliana accessions.

blast+ v2.9.0 (blastn and blastp) for sequence similarity search during homolog identification (default parameters).

GSTAr.pl v1.0 and psRNATarget (https://www.zhaolab.org/psRNATarget/) to calculate sRNA targeting scores on PPR transcripts.

Custom codon-based alignment tool (https://github.com/lyy005/codon_alignment) for CDS alignments in nucleotide diversity analysis.

R package Popgenome (sliding.window.transform function) for nucleotide diversity metrics (Pi, Watterson's theta).

R package APE(dist.dna function) for calculating genetic distances.

R package STATS (cor.test function) for correlation analysis (Spearman).

hmmsearch (PFAM HMM profiles) for identification of PPR proteins across proteomes.

Multiple sequence alignment tools: MUSCLE v3.8.31, trimAI v1.2rev59, FAMSA v2.2.2, MAFFT v7.525

Phylogenetic tree construction: IQ-TREE2 v2.0.4 with ultrafast bootstrapping (models VT+F+G4, LG+F+G4, JTTDCMut+I+G4) and FastTree v2.1.10/2.1.11 (LG model).

Tree visualization: R package ggtree v3.6.2 and iTOL online tool（https://itol.embl.de/）

Taxonomic tree: TimeTree 5 (timetree.org)

Statistical analyses: ANOVA followed by Tukey's HSD test were performed using JMP Pro v13.0 (SAS). The Kruskal–Wallis test with Bonferroni-adjusted post hoc comparisons was conducted using the R package agricolae.

For manuscripts utilizing custom algorithms or software that are central to the research but not yet described in published literature, software must be made available to editors and reviewers. We strongly encourage code deposition in a community repository (e.g. GitHub). See the Nature Portfolio guidelines for submitting code & software for further information.

Data

Policy information about availability of data

All manuscripts must include a data availability statement. This statement should provide the following information, where applicable:

- Accession codes, unique identifiers, or web links for publicly available datasets
- A description of any restrictions on data availability
- For clinical datasets or third party data, please ensure that the statement adheres to our policy

All data supporting the findings of this study are publicly available within the Article, its Supplementary Information, and in the specified public repositories. The raw sRNA-seq data of A. thaliana accession Col-0 are available in the NCBI SRA under accession code SRP135923 [https://www.ncbi.nlm.nih.gov/sra/?term=SRP135923]. Raw sRNA-seq data for the other seven A. thaliana accessions have been deposited in NCBI BioProject database under accession code PRJNA1037763 [https://www.ncbi.nlm.nih.gov/bioproject/PRJNA1037763]. Source data for all figures are provided. Source data are provided as supplementary files in this paper. All the original code is available at the GitHub [http://github.com/fionaliii/PPR-siRNA].

Research involving human participants, their data, or biological material

Policy information about studies with human participants or human data. See also policy information about sex, gender (identity/presentation), and sexual orientation and race, ethnicity and racism.

Reporting on sex and gender	N/A
Reporting on race, ethnicity, or other socially relevant groupings	N/A
Population characteristics	N/A
Recruitment	N/A
Ethics oversight	N/A

Note that full information on the approval of the study protocol must also be provided in the manuscript.

Field-specific reporting

Please select the one below that is the best fit for your research. If you are not sure, read the appropriate sections before making your selection.

[x] Life sciences [ ] Behavioural & social sciences [ ] Ecological, evolutionary & environmental sciences

For a reference copy of the document with all sections, see nature.com/documents/nr-reporting-summary-flat.pdf

Life sciences study design

All studies must disclose on these points even when the disclosure is negative.

Sample size	Sample size was determined based on experimental trials and previous publications on similar experiments. No statistical methods were used to predetermine sample size. Previous publications considered to determine sample size include:<br>Gene expression analysis (doi: 10.1126/science.aai8898)<br>Lesion length/area and fungal growth analysis (doi: 10.1126/science.aai8898; doi: 10.1016/j.molcel.2019.03.013.)
Data exclusions	No data were excluded from the analysis
Replication	All experiments were successfully repeated at least three times, and the number of independent experiments or biological replicates is indicated in the figure legends.
Randomization	Plants were randomly assigned to the treatment and control groups with no formal randomization techniques.
Blinding	The study primarily involves laboratory experiments rather than clinical trials, where blinding is more commonly applied. Blinding is not feasible in experiments involving plants or in vitro assays, as the nature of the materials and procedures does not lend itself to blinding. Researchers involved in conducting the experiments were aware of the experimental conditions and treatments due to the nature of the procedures, making blinding impractical. Blinding was not considered necessary given the objective and design of our study, which focused on elucidating molecular mechanisms rather than evaluating subjective outcomes. Blinding may introduce additional complexity and logistical challenges to experimental procedures without significantly improving the validity or reliability of the results in our experimental setup.

Reporting for specific materials, systems and methods

We require information from authors about some types of materials, experimental systems and methods used in many studies. Here, indicate whether each material, system or method listed is relevant to your study. If you are not sure if a list item applies to your research, read the appropriate section before selecting a response.

Materials & experimental systems

n/a	Involved in the study	
[ ]	[x]	Antibodies
[x]	[ ]	Eukaryotic cell lines
[x]	[ ]	Palaeontology and archaeology
[x]	[ ]	Animals and other organisms
[x]	[ ]	Clinical data
[x]	[ ]	Dual use research of concern
[ ]	[x]	Plants

Methods

n/a	Involved in the study	
[x]	[ ]	ChIP-seq
[x]	[ ]	Flow cytometry
[x]	[ ]	MRI-based neuroimaging

Antibodies

Antibodies used	anti-GFP (Clontech, Cat#632381, 1:2000)
Validation	Validation statements and experiments can be obtained from the following websites: https://www.takarabio.com/products/antibodies-and-elisa/fluorescent-protein-antibodies/green-fluorescent-protein-antibodies?catalog=632475

Dual use research of concern

Policy information about dual use research of concern

Hazards

Could the accidental, deliberate or reckless misuse of agents or technologies generated in the work, or the application of information presented in the manuscript, pose a threat to:

No	Yes	
☒	☐	Public health
☒	☐	National security
☒	☐	Crops and/or livestock
☒	☐	Ecosystems
☒	☐	Any other significant area

Experiments of concern

Does the work involve any of these experiments of concern:

No	Yes	
☒	☐	Demonstrate how to render a vaccine ineffective
☒	☐	Confer resistance to therapeutically useful antibiotics or antiviral agents
☒	☐	Enhance the virulence of a pathogen or render a nonpathogen virulent
☒	☐	Increase transmissibility of a pathogen
☒	☐	Alter the host range of a pathogen
☒	☐	Enable evasion of diagnostic/detection modalities
☒	☐	Enable the weaponization of a biological agent or toxin
☒	☐	Any other potentially harmful combination of experiments and agents

Plants

Seed stocks	Seeds of Arabidopsis for this study were ordered from the Arabidopsis Biological Resource Center (ABRC), including CS97808 (An-1), CS76786 (Ct-1), CS97810 (Cvi-1), CS97813 (Eri-1), CS97807 (Kyo-1), CS97814 (Ler-0), CS97811 (Sha), and CS737432 (62914mut). Transgenic Arabidopsis were generated in the lab.
Novel plant genotypes	To construct expression lines of AT1G62914 carrying a premature stop codon, the coding sequences of AT1G62914 from A. thaliana Col-0 was amplified using gene-specific primers. The PCR products were cloned into the pENTR/D-Topo vector and site-directed mutagenesis was performed to generate AT1G62914pm, which was cloned into the vector pEG104 using Gateway cloning (Invitrogen). To generate the AT1G62914del construct, two fragments of AT1G62914 flanking the deleted region were amplified and fused at the BsaI sites and the resulting fragments was cloned into pEG104 using In-Fusion cloning. Transgenic lines of Arabidopsis were generated by Agrobacterium-mediated transformation. Two independent transgenic lines at T3 generation were used for complementation experiments. To generate expression tobacco plants, Agrobacterium-mediated transient expression were performed by using Agroinfiltration. Agrobacteria harboring different vectors were mixed and infiltrated into the tobacco leaves to express sRNAs.
Authentication	Transgenic Arabidopsis were confirmed by the recovery of sRNA production.
