# Supplementary material for: A conserved small RNA-generating gene cluster undergoes sequence diversification and contributes to plant immunity
Source: Nat Commun. 2026 Aug 26;17:10187. doi: 10.1038/s41467-026-77164-1 (PMC13612597; doi:10.1038/s41467-026-77164-1)
Supplement: Supplementary file 2 [file 41467_2026_77164_MOESM14_ESM.pdf]

nature portfolio

Peer Review File

A conserved small RNA-generating gene cluster undergoes sequence diversification and contributes to plant immunity

Corresponding Author: Professor Wenbo Ma

This manuscript has been previously reviewed at another journal. This document only contains information relating to versions considered at Nature Communications.

This file contains all reviewer reports in order by version, followed by all author rebuttals in order by version.

Version 0:

Reviewer comments:

Reviewer #1

(Remarks to the Author)
The authors clearly demonstrate that a conserved clade of physically clustered PPRs is subjected to extensive sequence diversification in Arabidopsis as well as in other plant species. Furthermore, these siRNA-producing PPR genes show high-impact variations including pseudogenization. Importantly, the authors also provide evidence that siRNAs generated from an Arabidopsis pseudogene confers resistance against P. capsica in N. Benthamiana. This indicates that siRNAs derived from this pseudogene, which has likely lost its protein-coding capacity, confer resistance against this pathogen in a heterologous system. Because siRNA-producing PPRs are physically clustered, a genomic feature known to promote sequence diversification through gene duplication and rearrangements, the authors propose that the mechanisms governing sequence diversification at these loci may be similar to those observed at NLR clusters. This argues for a potential adaptive defensive role of PPRs, analogous to that observed at NLRs, whose extensive sequence diversification is thought to facilitate recognition of rapidly evolving pathogens. In my view, it is not surprising that a subset of PPRs can promote resistance against pathogens, whereas others function in reproductive functions. It is also possible that some of these PPRs would regulate both functions. Similarly, plant and mammalian NLRs were initially thought to function exclusively in microbe recognition; however, accumulating evidence now shows that they also regulate other biological processes.

Overall, I believe that this manuscript would be of broad interest to researchers working on plant immunity, RNA silencing, PPR biology, as well as cross-kingdom RNAi. Below are some constructive suggestions that I hope will help strengthen the discussion and provide complementary genomic evidence supporting a role for these PPRs in plant adaptation to pathogen threats. Of note, these additional genomic analyses, which could be performed within a relatively short timeframe, are suggested here as complementary analyses but are not mandatory for publication.

- Alternative evolutionary scenario that could be considered and discussed.

Although pathogen-associated selective pressures, together with reproductive strategies, may have contributed to the expansion and sequence diversification of these PPRs, it remains challenging to establish whether their emergence was initially driven by selection specifically for production of antimicrobial siRNAs. Instead, the production of secondary siRNAs might have primarily evolved as a mechanism to maintain expression homeostasis among duplicated PPR genes within the cluster, as described for phasiRNA-mediated regulation of NLRs. Under this scenario, trans-kingdom RNAi-based regulation mediated by PPR-derived siRNAs would have emerged subsequently as an additional function, further imposing a positive selective feedback regulation on the sequence diversification of the cluster, and ultimately leading to the emergence of pseudogenes with functional relevance in this process.

- A brief perspective on P. capsici genome evolution in the context of cross-kingdom RNAi would strengthen the discussion.

It would be helpful to discuss, as a perspective of this work, the need to study the natural sequence variation at sRNA targets across P. capsica genomes, particularly for targets of abundant plant-derived sRNAs produced by the main natural hosts of this pathogen. Such analyses could reveal whether P. capsici genomes have evolved to evade this regulatory mechanism or, conversely, to preserve it over the course of evolution. In the latter scenario, maintenance of these target sites could

confer a selective advantage by allowing plant-derived siRNAs to transiently restrict pathogen replication during specific phases of infection, thereby reducing its detection by the plant immune system, as well as the detrimental consequences of infection, such as premature host cell death. Conversely, during other stages, particularly at early phases of host invasion and colonization, during which effectors are generally more expressed, P. capsica could rely on its RNA silencing suppressors to alleviate this regulation and promote successful infection. Such a dynamic interplay between cross-kingdom RNAi and RNA silencing suppression would be consistent with the hemibiotrophic lifestyle of P. capsici.

- Additional genomic analyses to further support the authors’ hypothesis.

One important conclusion from the authors, which has been challenged by reviewer #2, is that the PPRs studied have evolved to confer disease resistance. This conclusion is notably supported by the genomic features of these loci, which are phylogenetically and physically clustered, and exhibit extensive sequence diversity and pseudogenization. These observations are partly analogous to those reported for NLRs, suggesting that these PPRs play a role in plant defense. To provide further genomic support for this hypothesis, I suggest analyzing whether clustered PRRs represent preferred integration sites for transposable elements (TEs)/repeats compared with non-siRNA producing PPRs or randomly selected genes. Such genomic feature is now well established for Arabidopsis NLRs, which exhibit a high propensity for TE and repeat insertions in their vicinity (e.g. promoters), as well as inside introns, or even exons, during somatic transposition events (e.g., PMID: 42401980), and as a result of evolution (e.g., PMID: 40966307, PMID: 33957946, PMID: 29871888, PMID: 33470193, PMID: 23940361). If these PPRs similarly act as sponges of TEs/repeats, these findings would provide further support for their role in both adaptation to pathogen threats, and sequence diversification.

(Remarks on code availability)

Open Access This Peer Review File is licensed under a Creative Commons Attribution 4.0 International License, which permits use, sharing, adaptation, distribution and reproduction in any medium or format, as long as you give appropriate credit to the original author(s) and the source, provide a link to the Creative Commons license, and indicate if changes were made.
In cases where reviewers are anonymous, credit should be given to 'Anonymous Referee' and the source.
The images or other third party material in this Peer Review File are included in the article’s Creative Commons license, unless indicated otherwise in a credit line to the material. If material is not included in the article’s Creative Commons license and your intended use is not permitted by statutory regulation or exceeds the permitted use, you will need to obtain permission directly from the copyright holder.
To view a copy of this license, visit https://creativecommons.org/licenses/by/4.0/

Editor's comments

In particular, this reviewer suggests to consider alternative evolutionary scenarios for the emergence of siRNA-producing PPRs and to expand the discussion to include the evolutionary dynamics of P. capsici target sites and the potential interplay between cross-kingdom RNAi and pathogen RNA-silencing suppression. Editorially we believe that addressing these discussion points would improve the manuscript.

Response: We appreciate the guidance from the editor. The two points raised by reviewer 1 have been incorporated in the discussion session.

Reviewer #1 (Remarks to the Author):

The authors clearly demonstrate that a conserved clade of physically clustered PPRs is subjected to extensive sequence diversification in Arabidopsis as well as in other plant species. Furthermore, these siRNA-producing PPR genes show high-impact variations including pseudogenization. Importantly, the authors also provide evidence that siRNAs generated from an Arabidopsis pseudogene confers resistance against P. capsica in N. Benthamiana. This indicates that siRNAs derived from this pseudogene, which has likely lost its protein-coding capacity, confer resistance against this pathogen in a heterologous system. Because siRNA-producing PPRs are physically clustered, a genomic feature known to promote sequence diversification through gene duplication and rearrangements, the authors propose that the mechanisms governing sequence diversification at these loci may be similar to those observed at NLR clusters. This argues for a potential adaptive defensive role of PPRs, analogous to that observed at NLRs, whose extensive sequence diversification is thought to facilitate recognition of rapidly evolving pathogens. In my view, it is not surprising that a subset of PPRs can promote resistance against pathogens, whereas others function in reproductive functions. It is also possible that some of these PPRs would regulate both functions. Similarly, plant and mammalian NLRs were initially thought to function exclusively in microbe recognition; however, accumulating evidence now shows that they also regulate other biological processes.

Overall, I believe that this manuscript would be of broad interest to researchers working on plant immunity, RNA silencing, PPR biology, as well as cross-kingdom RNAi. Below are some constructive suggestions that I hope will help strengthen the discussion and provide complementary genomic evidence supporting a role for these PPRs in plant adaptation to pathogen threats. Of note, these additional genomic analyses, which could be performed within a relatively short timeframe, are suggested here as complementary analyses but are not mandatory for publication.

Response: We thank the reviewer for the overall positive feedback and constructive suggestions.

- Alternative evolutionary scenario that could be considered and discussed.

Although pathogen-associated selective pressures, together with reproductive strategies, may have contributed to the expansion and sequence diversification of these PPRs, it remains challenging to establish whether their emergence was initially driven by selection specifically for production of antimicrobial siRNAs. Instead, the production of secondary siRNAs might have primarily evolved as a mechanism to maintain expression homeostasis among duplicated PPR genes within the cluster, as described for phasiRNA-mediated regulation of NLRs. Under this scenario, trans-kingdom RNAi-based regulation mediated by PPR-derived siRNAs would have emerged subsequently as an additional function, further imposing a positive selective feedback regulation on the sequence diversification of the cluster, and ultimately leading to the emergence of pseudogenes with functional relevance in this process.

Response: We have added a discussion in line 422-432 to address this point.

- A brief perspective on P. capsici genome evolution in the context of cross-kingdom RNAi would strengthen the discussion.

It would be helpful to discuss, as a perspective of this work, the need to study the natural sequence variation at sRNA targets across P. capsica genomes, particularly for targets of abundant plant-derived sRNAs produced by the main natural hosts of this pathogen. Such analyses could reveal whether P. capsici genomes have evolved to evade this regulatory mechanism or, conversely, to preserve it over the course of evolution. In the latter scenario, maintenance of these target sites could confer a selective advantage by allowing plant-derived siRNAs to transiently restrict pathogen replication during specific phases of infection, thereby reducing its detection by the plant immune system, as well as the detrimental consequences of infection, such as premature host cell death. Conversely, during other stages, particularly at early phases of host invasion and colonization, during which effectors are generally more expressed, P. capsica could rely on its RNA silencing suppressors to alleviate this regulation and promote successful infection. Such a dynamic interplay between cross-kingdom RNAi and RNA silencing suppression would be consistent with the hemibiotrophic lifestyle of P. capsici.

Response: We have added a discussion emphasizing the need of understanding the evolutionary dynamics of pathogen target genes as a future research direction in line

460-463. The rest of the reviewer's suggestion about discussing siRNA-based defense at specific pathogen infection phases, in our opinion, seems to be a bit speculative and may not be ready to be discussed in the current study. We propose to include this discussion in future publication when further evidence can be provided.

- Additional genomic analyses to further support the authors' hypothesis.

One important conclusion from the authors, which has been challenged by reviewer #2, is that the PPRs studied have evolved to confer disease resistance. This conclusion is notably supported by the genomic features of these loci, which are phylogenetically and physically clustered, and exhibit extensive sequence diversity and pseudogenization. These observations are partly analogous to those reported for NLRs, suggesting that these PPRs play a role in plant defense. To provide further genomic support for this hypothesis, I suggest analyzing whether clustered PRRs represent preferred integration sites for transposable elements (TEs)/repeats compared with non-siRNA producing PPRs or randomly selected genes. Such genomic feature is now well established for Arabidopsis NLRs, which exhibit a high propensity for TE and repeat insertions in their vicinity (e.g. promoters), as well as inside introns, or even exons, during somatic transposition events (e.g., PMID: 42401980), and as a result of evolution (e.g., PMID: 40966307, PMID: 33957946, PMID: 29871888, PMID: 33470193, PMID: 23940361). If these PPRs similarly act as sponges of TEs/repeats, these findings would provide further support for their role in both adaptation to pathogen threats, and sequence diversification.

Response: We agree with the reviewer that this would be a great analysis and are grateful for their understanding that this analysis is not mandatory for publication.
