# Supplementary material for: A conserved small RNA-generating gene cluster undergoes sequence diversification and contributes to plant immunity
Source: Nat Commun. 2026 Aug 26;17:10187. doi: 10.1038/s41467-026-77164-1 (PMC13612597; doi:10.1038/s41467-026-77164-1)
Supplement: Supplementary file 3 [file 41467_2026_77164_MOESM1_ESM.pdf]

Title page:

A conserved small RNA-generating gene cluster undergoes sequence diversification and contributes to plant immunity

Li Feng[1,#], Yingnan Hou[2,#,*], AmirAli Toghani[1], Zhixue Wang[3], Bozeng Tang[1], Nicola Atkinson[1], Hui Li[1], Yue Qiao[2], Yan Wang[4], Jian Hua[3], Jixian Zhai[5], Wenbo Ma[1,*]

[1] The Sainsbury Laboratory, University of East Anglia, Norwich Research Park, Norwich, NR4 7UH, UK

[2] Shanghai Collaborative Innovation Center of Agri-Seeds/School of Agriculture & Biology, Shanghai Jiao Tong University, Shanghai 200240, China

[3] School of Integrative Plant Science Plant Biology Section, Cornell University, Ithaca, NY 14853, USA

[4] Department of Biological Science, University of Toronto, Scarborough, Ontario, M1C 1A4, Canada

[5] Institute of Plant and Food Science, Department of Biology, Southern University of Science and Technology, Shenzhen 518055, China

* Correspondence: Wenbo Ma (wenbo.ma@tsl.ac.uk) or Yingnan Hou (yingnanh@sjtu.edu.cn)

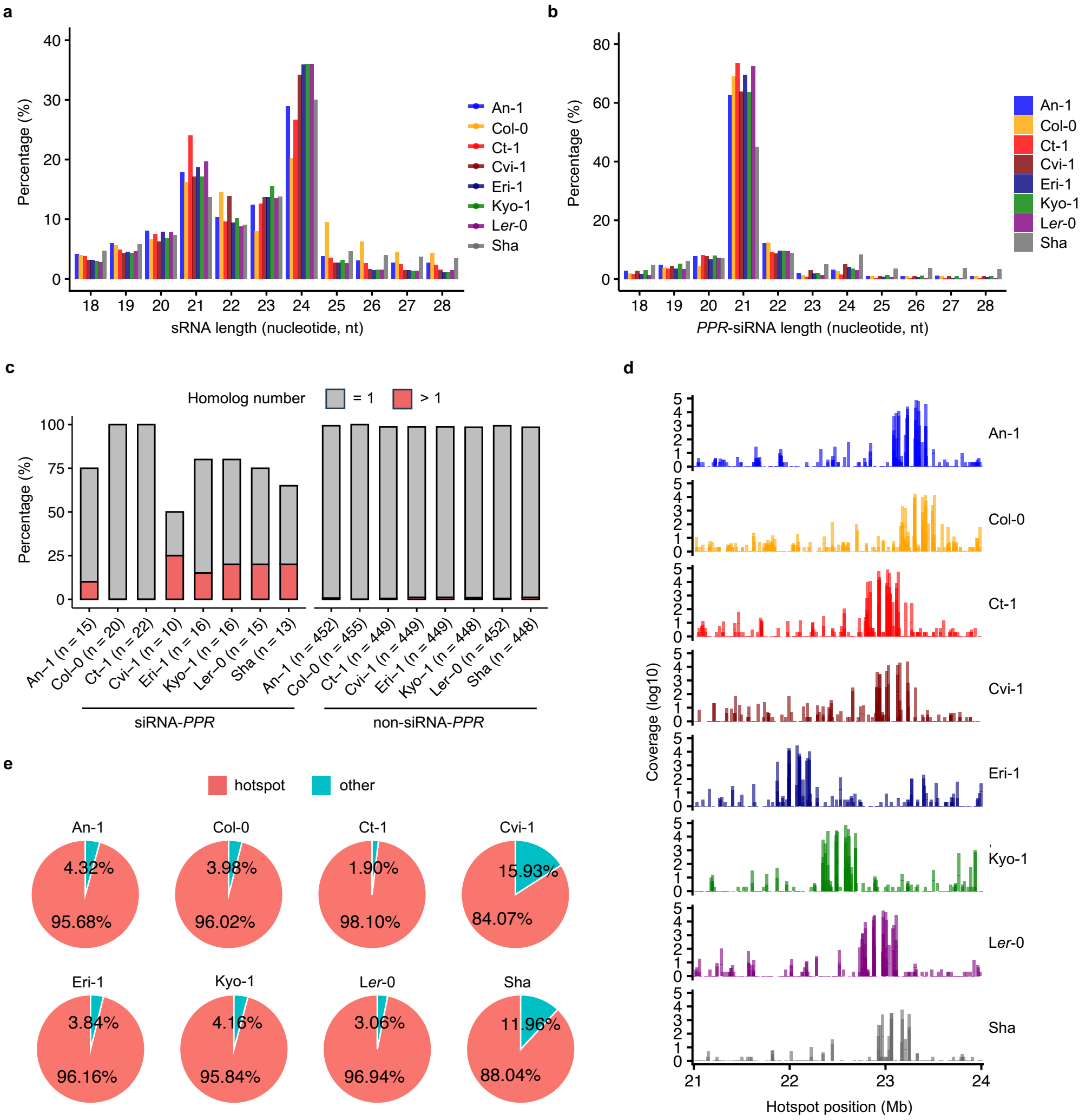

Figure S1. sRNA-seq analysis of *PPR*-siRNAs in eight *A. thaliana* accessions.

a. Size distribution of the total sRNA population in each *A. thaliana* accession. **b.** Size distribution of *PPR*-siRNAs in each *A. thaliana* accession. **c.** Percentage of siRNA-producing *PPR* genes (TPM > 5) and non-siRNA-producing *PPR* genes (TPM ≤ 5) in Col-0 that have one or more homologs in each of the other accessions. Numbers in parentheses indicate the number of *PPR* genes. **d.** Zoomed-in view of the siRNA-generating hotspot region on Chromosome 1. **e.** Percentage of siRNAs produced from hotspot siRNA-producing *PPR* genes in each accession. A Source Data file is also provided.

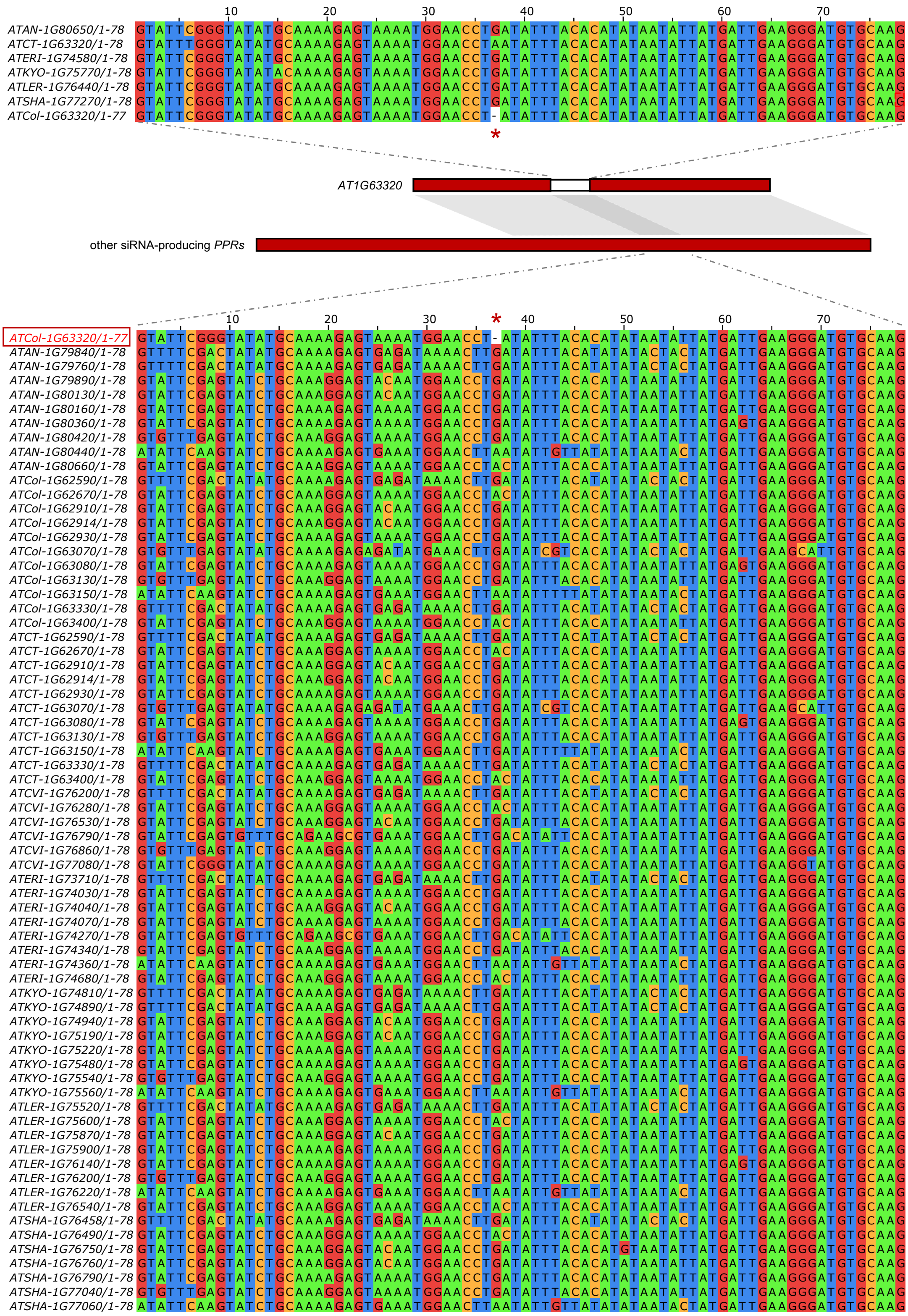

Figure S2. ***AT1G63320*** **contains a single intron that is present as exon sequences of other siRNA-producing** ***PPR*****s located in the hotspot cluster.**

Top: Sequence alignment of the single intron from *AT1G63320* homologs across seven *A. thaliana* accessions. The *AT1G63320* intron is 77 nt in Col-0 and 78 nt in the other accessions. Red asterisk indicates the nucleotide gap in Col-0. Middle: Gene models of *AT1G63320* in Col-0 as a representative of the other siRNA-producing *PRR* genes. Exons are shown as red boxes and the intron as a white box. Grey shading indicates regions with sequence similarity. Bottom: Alignment of the Col-0 *AT1G63320* intron sequence to the corresponding CDS region of 68 siRNA-producing *PPR* genes in the G1 group, which contain a 78 nt-region with high sequence similarity within the exon. A Source Data file is also provided.

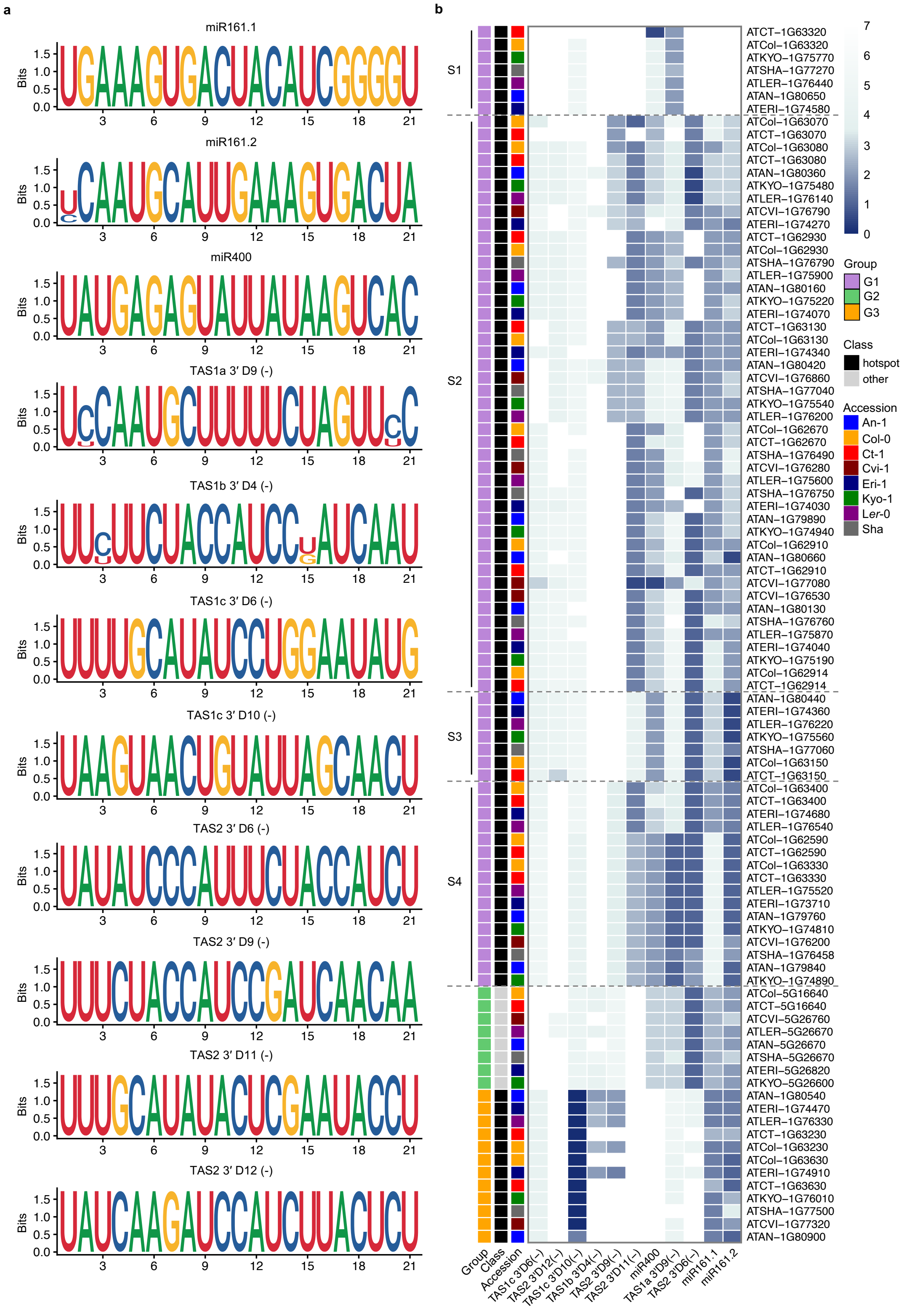

Figure S3. Target site analysis of siRNA-producing *PPRs* in *A. thaliana*.

a. sRNAs triggering *PPR*-siRNA production are highly conserved across eight *A. thaliana* accessions. Sequence logos were generated for 11 sRNAs that have been demonstrated to trigger *PPR*-siRNA

production using homologous sequences identified in the eight accessions. The height of each nucleotide indicates its relative frequency at that position, and the total height of each stack reflects the degree of sequence conservation. **b.** Predicted target sites of the 11 sRNA triggers in siRNA-producing *PPR* genes. Heatmap colors represent the targeting expectation score of each sRNA trigger for each gene (lower scores indicate higher targeting specificity). A Source Data file is also provided.

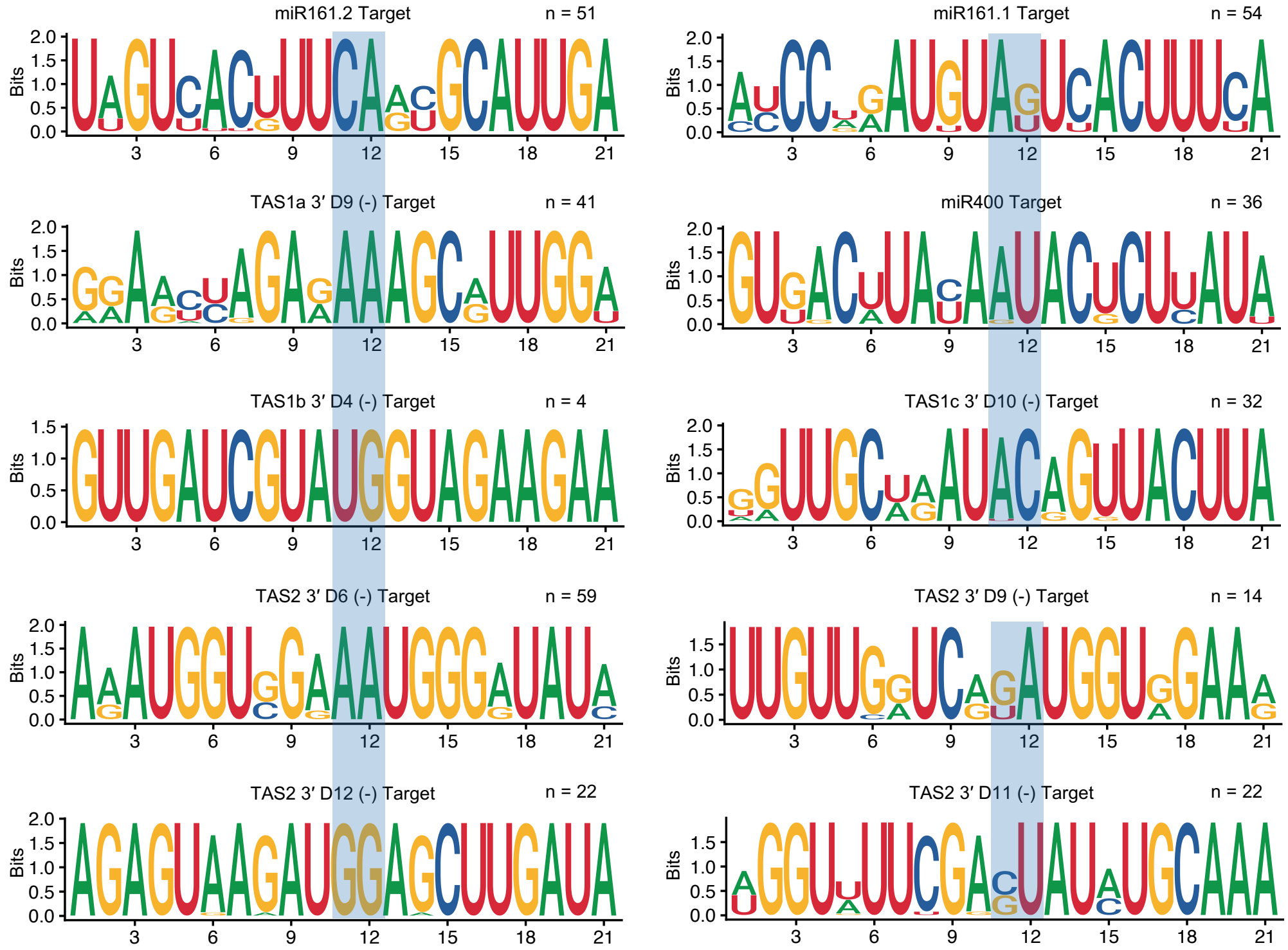

Figure S4. Conservation of trigger sRNA target sites in siRNA-producing *PPR*s.

Conservation of predicted trigger sRNA target sites in 115 conserved siRNA-producing *PPR* genes (indicated in Figure 1a) across eight *A. thaliana* accessions. Sequence logos were generated using multiple alignments of the predicted target sites (AllenScore < 4). The number of predicted target sites analyzed for each trigger sRNA is indicated as *n*. The height of each nucleotide indicates relative frequency at that position, and the total height of each stack reflects the degree of sequence conservation. The blue shading highlights the 10-11 (5′-3′) positions of each trigger sRNA, which correspond to the canonical cleavage site and are considered critical for target recognition. A Source Data file is also provided.

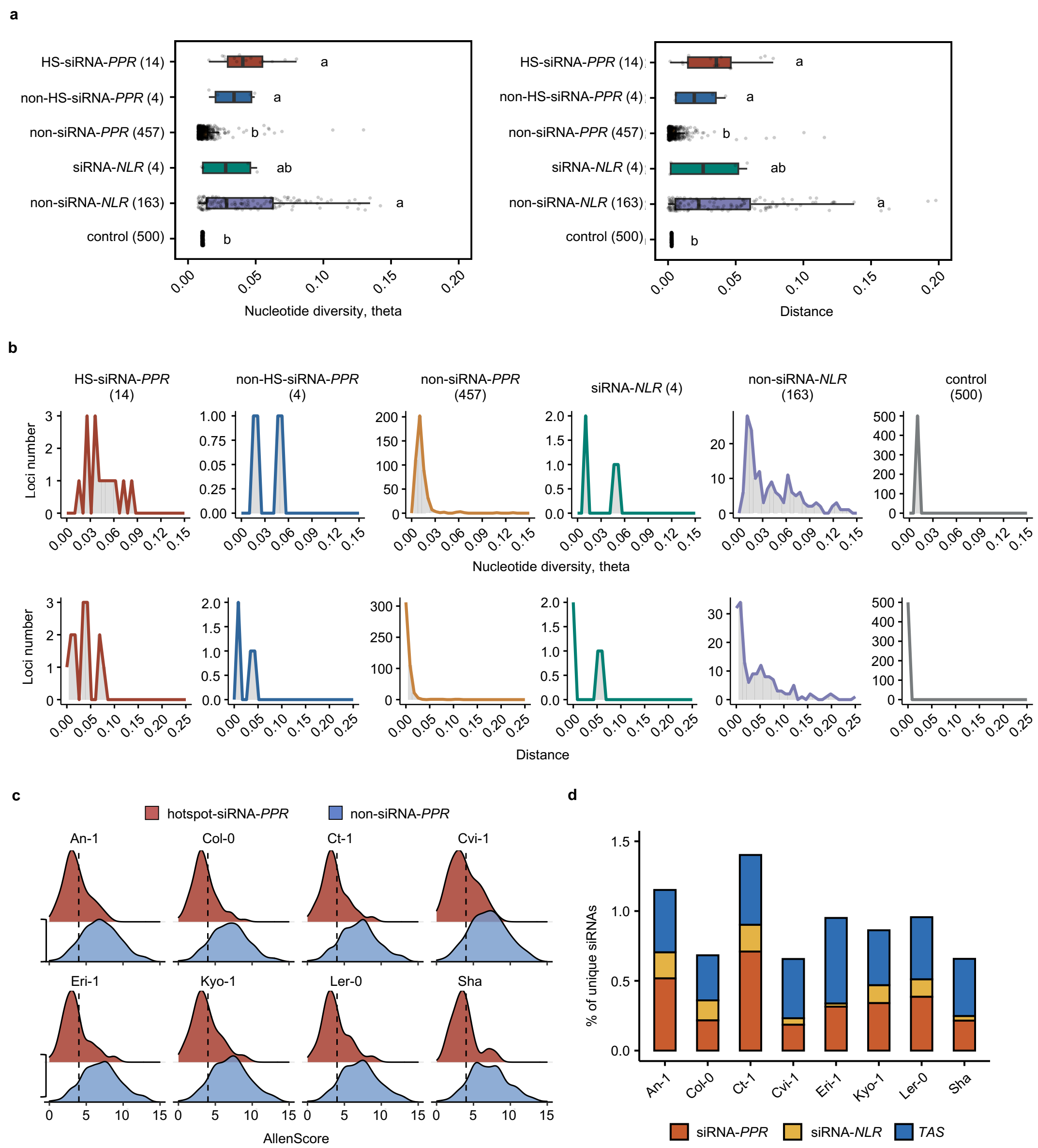

Figure S5. Sequence diversity analysis of siRNA-producing *PPR*s.

a. Nucleotide diversity across six gene groups determined by Watterson's theta (Left) and Distance (Right) using the coding sequences from eight *A. thaliana* accessions. Numbers in parentheses indicate the number of genes in each group in Col-0. HS, hotspot. **b**. Distribution of Watterson's theta and Distance values for the six gene groups. **c**. Distribution of AllenScore, reflecting the predicted targeting ability of sRNA triggers, in the hotspot-siRNA-producing *PPR* genes and non-siRNA-*PPR* genes in each *A. thaliana* accession. Consistently lower AllenScores in hotspot-siRNA-producing *PPR*s indicate a conserved higher potential to be targeted by the trigger sRNAs. **d**. Contribution of *PPR*, *NLR* and *TAS* genes to the overall diversity of the 21-nt siRNA population in the eight *A. thaliana* accessions. Proportion of unique sequences of 21-nt siRNAs originating from these loci in each accession was calculated relative to the total number of unique 21-nt siRNAs in the same accession. For box plots in (a), center lines represent the median (50th percentile), box limits show the 25th and 75th percentiles (interquartile range, IQR), and whiskers extend to the minima and maxima values within 1.5 x IQR. Individual points represent single genes. Statistically significant differences were evaluated using a non-parametric Kruskal-Wallis test with Bonferroni-adjusted post hoc comparisons and labeled by different letters (adjusted $P < 0.05$). Adjusted P values and test statistics are provided in the Source Data file.

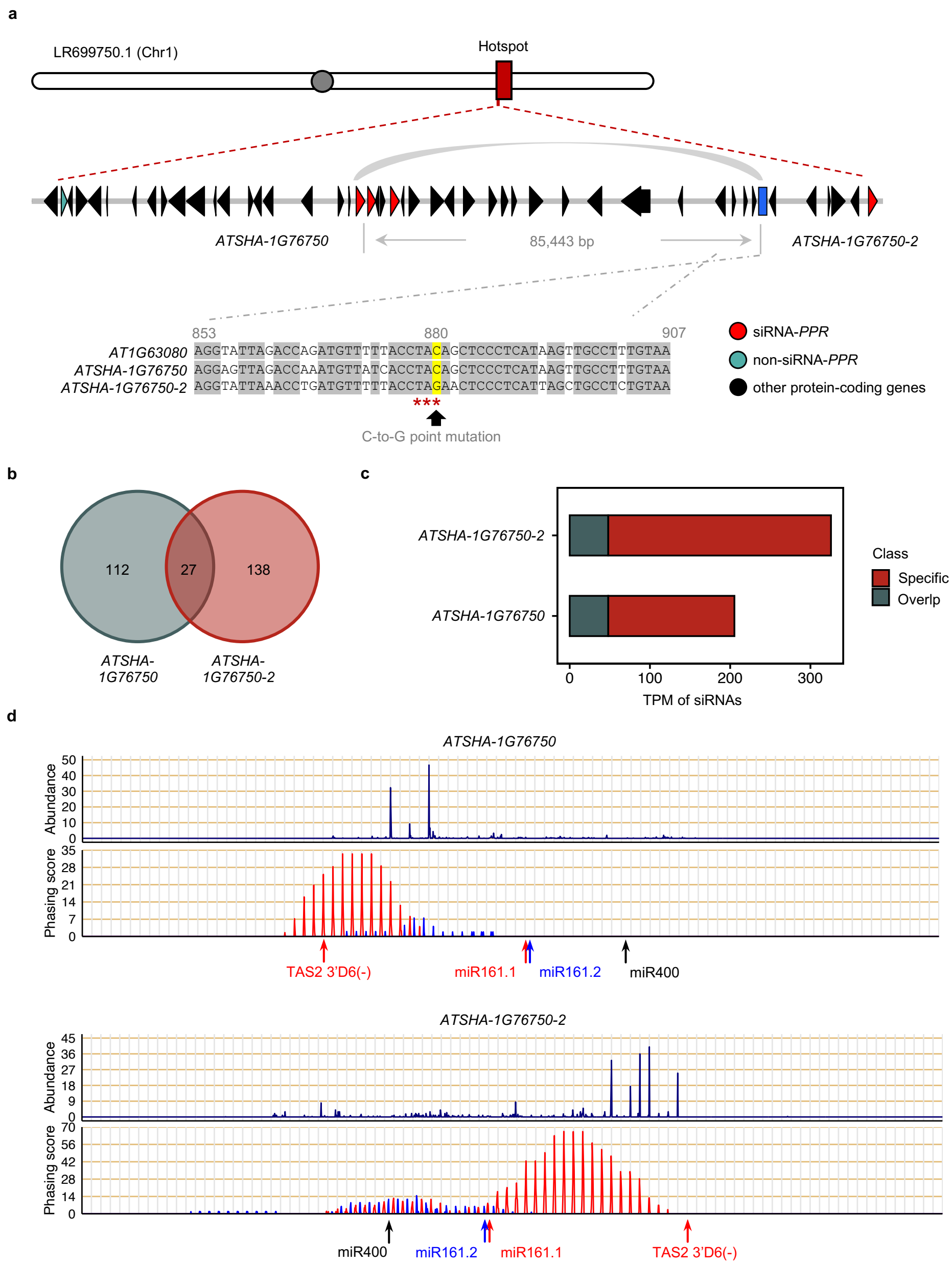

Figure S6. Pseudogenization of a hotspot-siRNA-producing *PPR* in the accession Sha.

a. Pseudogenization of a siRNA-producing *PPR* in Sha. A region within the *PPR*-siRNA hotspot cluster in Chromosome 1 contains two homologs of the Col-0 *PPR AT1G63080*. Red, cyan, and black triangles represent siRNA-producing *PPR*, non-siRNA-producing *PPR*, and non-*PPR* genes, respectively. One of the homologs, *ATSHA-1G76750*, is predicted to encode a PPR protein, while the other, *ATSHA-1G76750-2*, is annotated as a pseudogene located in an intergenic region (the blue rectangle). Sequence alignment shows a C-to-G mutation at the nucleotide position 922 that introduces a premature stop codon in *ATSHA-1G76750-2*. **b.** A limited number of 21-nt siRNAs produced from both *ATSHA-1G76750* and *ATSHA-1G76750-2*. **c.** The majority of siRNAs (represented by TPM) are uniquely produced from either *ATSHA-1G76750* or *ATSHA-1G76750-2*. **d.** Phasing patterns of siRNAs produced from *ATSHA-1G76750* and *ATSHA-1G76750-2*. Arrowheads indicate the cleavage sites of sRNA triggers. The colors of the arrowheads, i.e., red and blue, correspond to the red and blue phasing patterns of the siRNAs, respectively, which are conserved between the two homologs. Black arrowheads

indicate target sites that are not associated with phasing patterns of the siRNAs. A Source Data file is also provided.

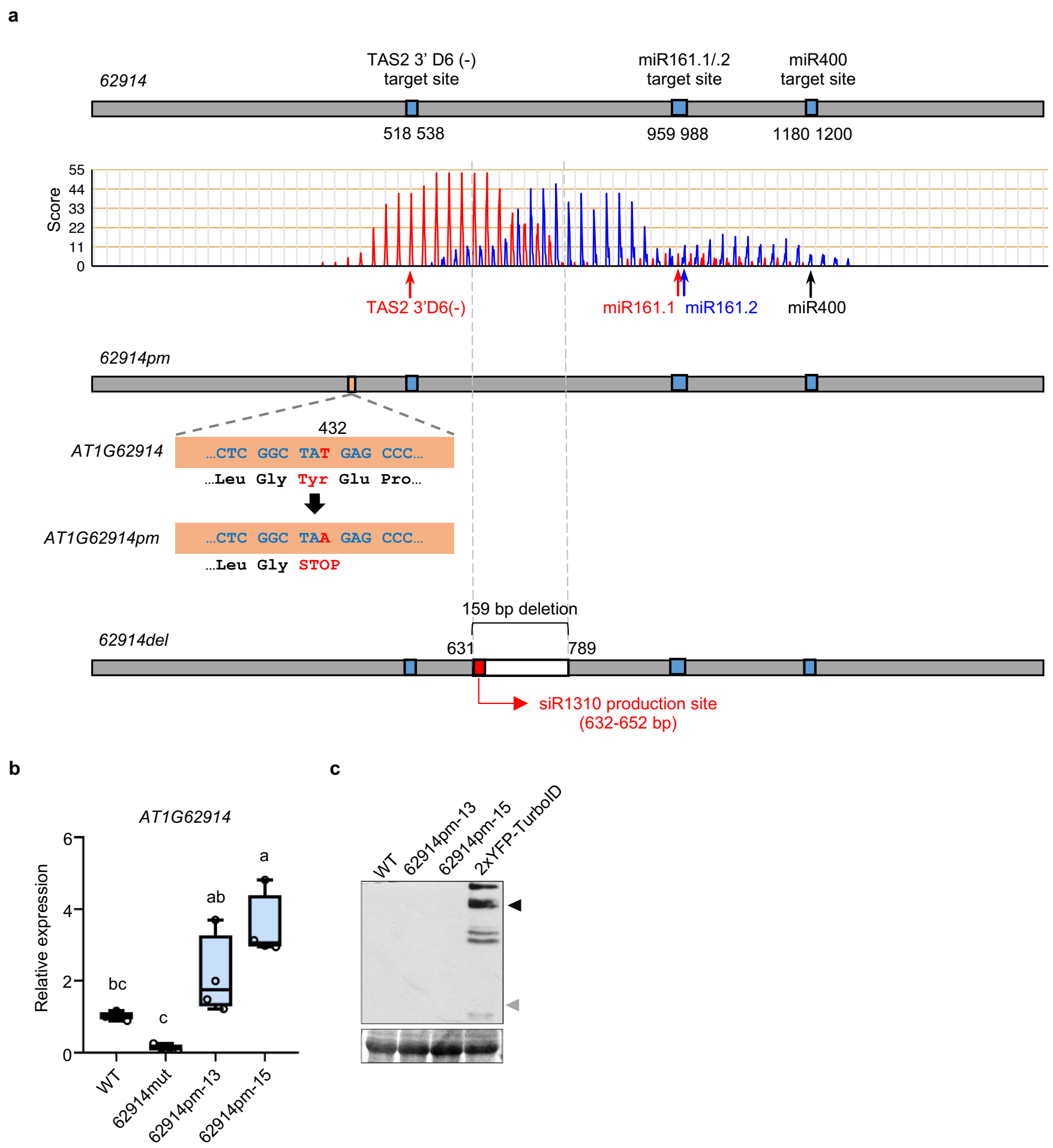

Figure S7. Construction of *AT1G62914* mutants for investigating siRNA production and defense activity in pseudogenized *PPR* genes.

a. A schematic of *AT1G62914* with predicted trigger sRNA target sites (indicated as blue rectangles) and associated siRNA production. Phased siRNA production from the *AT1G62914* locus is visualized by the phasing register pattern. Arrowheads indicate the cleavage sites of sRNA triggers. Red and blue arrows correspond to the red and blue phasing patterns of the siRNAs, respectively. The black arrowhead indicates a predicted target site that is not associated with any phasing pattern. Bottom**:** Schematics of *AT1G62914pm* and *AT1G62914del*. *AT1G62914pm* carries a single nucleotide mutation (highlighted in orange) in *AT1G62914* that introduces a premature stop codon. *AT1G62914del* contains a 159-bp deletion (indicated by the white rectangle), which eliminates the production of siR1310 without affecting the trigger sRNA target sites. The region producing siR1310 is highlighted by a red rectangle. **b.** Relative transcript abundance of full-length *AT1G62914* in *62914mut* (a T-DNA insertion mutant of *AT1G62914*) and two independent complementation lines (*62914pm-13* and *62914pm-15*) expressing *AT1G62914pm*. Data are presented as mean ± SD (n = 4 independent biological replicates). Statistically significant differences were evaluated using one-way ANOVA with Tukey's post hoc test and labeled by different letters ($P < 0.05$). **c.** Protein products were undetectable in *62914pm-13* and *62914pm-15* plants by western blotting using an anti-GFP antibody. Black and grey arrowheads indicate the expected sizes of potential YFP-AT1G62914 (about 85 kDa) and YFP-62914pm protein products (about 42 kDa), respectively. Adjusted P values and test statistics are provided in the Source Data file.

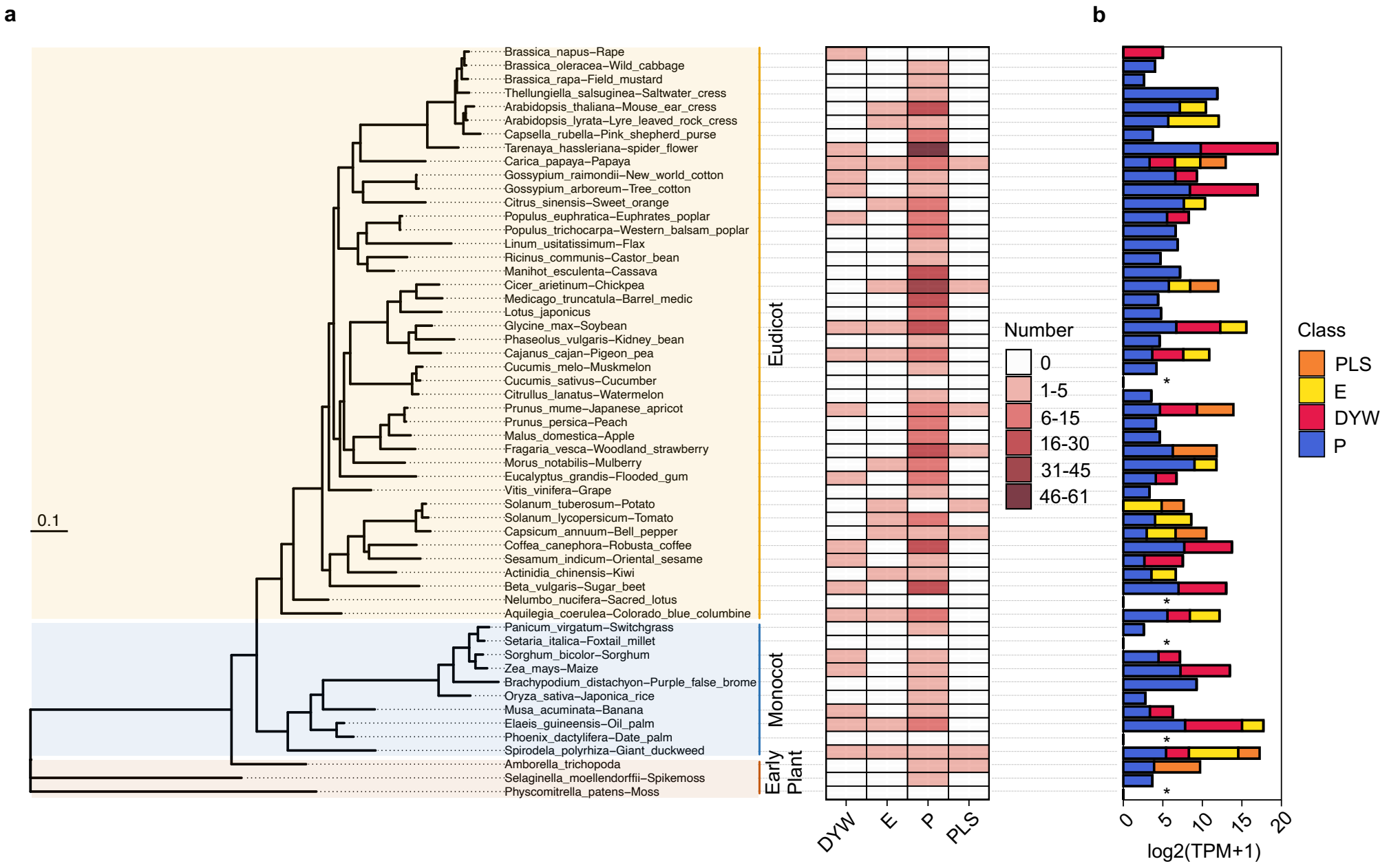

Figure S8. *PPR*-siRNA production is a common phenomenon across diverse plant species.

a. siRNA-producing *PPR*s were analyzed in 55 plant species, including three early plants, ten monocots, and 42 eudicots. A phylogenetic tree was constructed using the amino acid sequences of the mitochondrial transcription termination factor (AT2G21710 in *A. thaliana*). The heatmap shows the number of siRNA-producing loci in each of the DYW, E, P, and PLS classes of PPR proteins. **b.** Genes encoding P-class PPRs are major sources of siRNA production in plants. The barplot shows the abundance of *PPR*-siRNAs derived from genes belonging to different PPR classes. Values represent the average $\log_2$-transformed TPM of all the gene members within each class. A Source Data file is also provided.

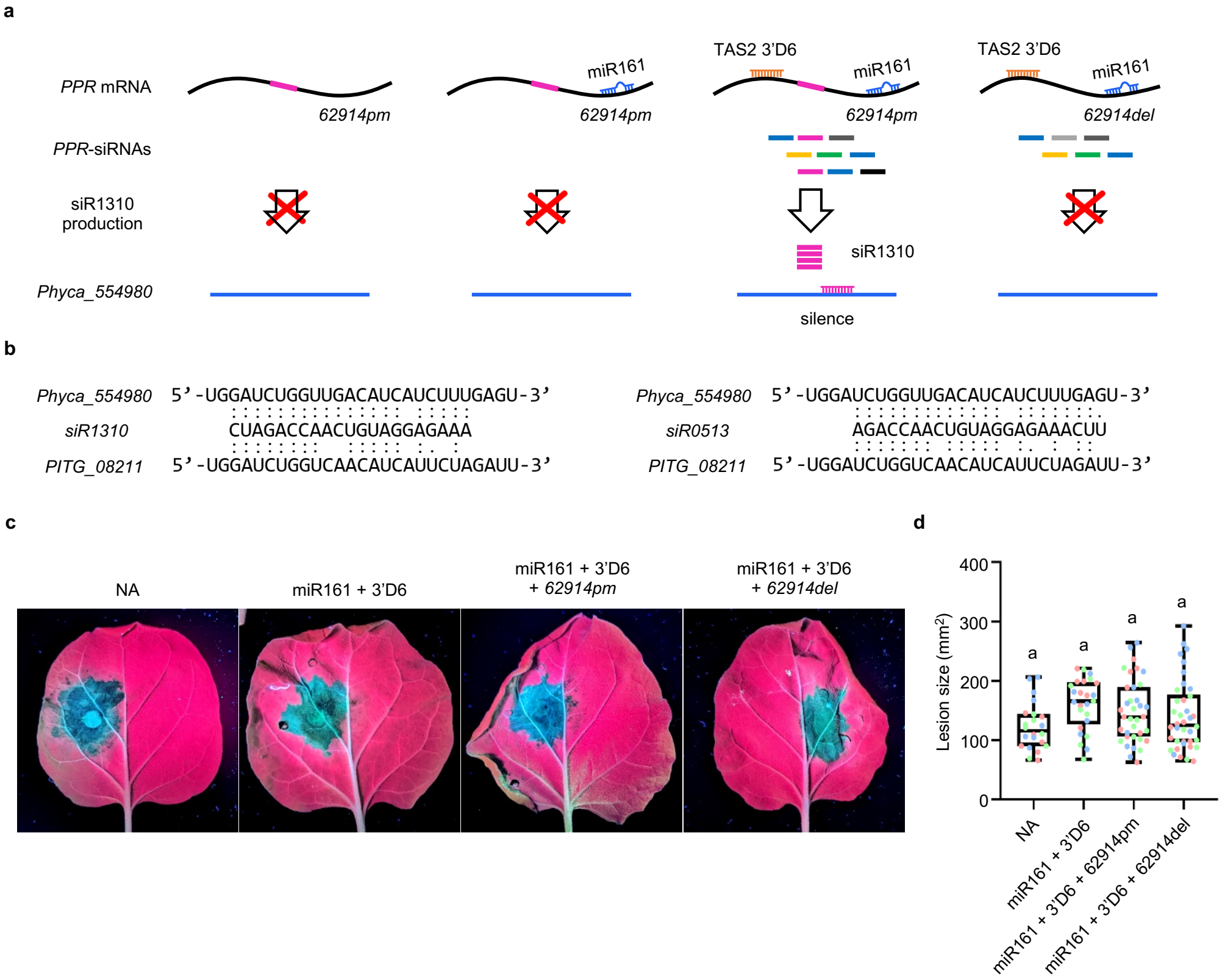

Figure S9. Ectopic production of a *PPR*-siRNA-producing cassette in *N. benthamiana* did not affect the resistance to *Phytophthora infestans*.

a. Construction of mutants of the *A. thaliana PPR* gene *AT1G62914* and their predicted impact on siR1310 production. **b**. *AT1G62914*-derived siRNAs can target the *P. capsici* gene *Phyca_554980* but not its homolog in *P. infestans*. Base pairing between *AT1G62914*-derived siRNAs, siR1310 and siR0513, and the *Phyca_554980* homologs in *P. capsici* and *P. infestans* (*PITG_08211*). **c**. Lesions formed on *N. benthamiana* leaves after inoculation with *P. infestans* at 5 dpi. **d.** Lesion sizes measured on inoculated *N. benthamiana* leaves at 5 dpi ($n \geq 7$ leaves per treatment across three independent replicates). For the box plot, center lines represent the median (50th percentile), box limits show the 25th and 75th percentiles (IQR), and whiskers extend to minima and maxima within 1.5 x IQR. Each data point represents an individual leaf. Statistically significant differences were evaluated using one-way ANOVA with Tukey's post hoc test and labeled by different letters ($P < 0.05$). Adjusted P values and test statistics are provided in the Source Data file.
