# Supplementary material for: A conserved small RNA-generating gene cluster undergoes sequence diversification and contributes to plant immunity
Source: Nat Commun. 2026 Aug 26;17:10187. doi: 10.1038/s41467-026-77164-1 (PMC13612597; doi:10.1038/s41467-026-77164-1)
Supplement: Supplementary file 4 [file 41467_2026_77164_MOESM2_ESM.pdf]

Title page:

A conserved small RNA-generating gene cluster undergoes sequence diversification and contributes to plant immunity

Li Feng[1,#], Yingnan Hou[2,#,*], AmirAli Toghani[1], Zhixue Wang[3], Bozeng Tang[1], Nicola Atkinson[1], Hui Li[1], Yue Qiao[2], Yan Wang[4], Jian Hua[3], Jixian Zhai[5], Wenbo Ma[1,*]

[1] The Sainsbury Laboratory, University of East Anglia, Norwich Research Park, Norwich, NR4 7UH, UK

[2] Shanghai Collaborative Innovation Center of Agri-Seeds/School of Agriculture & Biology, Shanghai Jiao Tong University, Shanghai 200240, China

[3] School of Integrative Plant Science Plant Biology Section, Cornell University, Ithaca, NY 14853, USA

[4] Department of Biological Science, University of Toronto, Scarborough, Ontario, M1C 1A4, Canada

[5] Institute of Plant and Food Science, Department of Biology, Southern University of Science and Technology, Shenzhen 518055, China

* Correspondence: Wenbo Ma (wenbo.ma@tsl.ac.uk) or Yingnan Hou (yingnanh@sjtu.edu.cn)

Supplementary Data 1

Title: Summary of raw, processed, and mapped reads for sRNA-seq libraries.

Legend: Detailed sequencing and data-filtering statistics for all sRNA-seq libraries. For each library, total and unique read counts are provided across successive processing steps, including raw reads, adapter-trimmed reads, quality-controlled reads, 18- to 28-nt reads mapped to the reference genome, reads derived from non-coding RNAs (tRNAs, rRNAs, snRNAs, and snoRNAs), and final filtered clean sRNA reads.

Supplementary Data 2

Title: Homologous PPR, NLR, and TAS loci identified across eight Arabidopsis thaliana accessions.

Legend: Genomic coordinates and annotation details of homologous gene loci identified in eight Arabidopsis thaliana accessions, provided in three separate sheets:

- **Sheet 1 (PPR loci):** Homologous pentatricopeptide repeat (PPR) loci**.**
- **Sheet 2 (NLR loci):** Homologous nucleotide-binding leucine-rich repeat (NLR) disease resistance loci.
- **Sheet 3 (TAS loci):** Homologous trans-acting siRNA (TAS) loci.

Supplementary Data 3

Title: Abundance, distribution, and evolutionary conservation of PPR-derived siRNAs across eight Arabidopsis thaliana accessions.

Legend: Comprehensive profiling and classification of PPR-derived small interfering RNAs (PPR-siRNAs) in eight Arabidopsis thaliana accessions, provided across seven separate sheets:

- **Sheet 1 (PPR-siRNA TPM):** Normalized abundance (TPM) of PPR-siRNAs across eight accessions.
- **Sheet 2 (Expressed siRNA-PPRs):** List of expressed siRNA-producing PPR loci (TPM > 5).
- **Sheet 3 (Conserved siRNA-PPRs):** List of conserved siRNA-producing PPR loci.
- **Sheet 4 (Hotspot siRNA-PPRs):** List of hotspot siRNA-producing PPR loci.
- **Sheet 5 (Clusters and Clades):** Detailed breakdown of siRNA-PPRs and conserved siRNA-PPRs in hotspot clusters and phylogenetic clades.
- **Sheet 6 (Homolog Counts):** Counts of siRNA-producing and non-siRNA-producing PPR homologs across accessions.

- **Sheet 7 (Proportions):** Proportion of PPR-siRNAs generated from hotspot clusters or phylogenetic clades.

Supplementary Data 4

Title: Sequences of sRNA triggers and predicted sRNA-target interactions in eight Arabidopsis thaliana accessions.

Legend: Comprehensive dataset containing sequences of sRNA triggers targeting PPR transcripts and predicted sRNA-target interactions across eight Arabidopsis thaliana accessions, provided in 21 separate sheets:

- **Sheet 1 (sRNA Triggers):** Sequences and homologs of sRNA triggers across eight accessions.
- **Sheets 2–10 (psRNATarget - PPRs):** Predicted sRNA-PPR target interactions identified by psRNATarget, provided for all eight accessions combined (**Sheet 2**) and individually for each accession (An-1, Col-0, Ct-1, Cvi-1, Eri-1, Kyo-1, Ler-0, Sha; **Sheets 3–10**).
- **Sheets 11–19 (GSTAr - PPRs):** Predicted sRNA-PPR target interactions identified by GSTAr (v1.0), provided for all eight accessions combined (**Sheet 11**) and individually for each accession (**Sheets 12–19**).
- **Sheet 20 (psRNATarget - Pseudogenes):** Predicted sRNA-pseudogene interactions identified by psRNATarget.
- **Sheet 21 (GSTAr - Pseudogenes):** Predicted sRNA-pseudogene interactions identified by GSTAr (v1.0).

Supplementary Data 5

Title: Nucleotide sequences of wild-type and mutant AT1G62914 constructs.

Legend: Full-length nucleotide sequences of wild-type AT1G62914, point-mutated construct (AT1G62914pm), and deletion construct (AT1G62914del) used in this study.

Supplementary Data 6

Title: Genome sources and siRNA expression profiles of PPR subfamilies across plant species.

Legend: Genomic resource information and small RNA expression profiles for plant species analyzed in this study, provided in two separate sheets:

- **Sheet 1 (Genome Sources):** Summary of genome assembly sources and database links for all plant species analyzed in this study.

- **Sheet 2 (PPR Subfamily siRNA TPM):** Normalized abundance (TPM) of siRNAs derived from individual PPR subfamily members across analyzed plant species.

Supplementary Data 7

Title: Metadata and quality metrics for 17 RefSeq proteomes used in PPR phylogenetic analysis.

Legend: Detailed assembly metadata, annotation statistics, BUSCO completeness scores, and taxonomic classification for the 17 RefSeq proteomes analyzed in the PPR phylogenetic reconstruction.

Supplementary Data 8

Title: Identification, filtering, and phylogenetic classification of PPR proteins across 17 RefSeq proteomes.

Legend: Comprehensive dataset of PPR proteins identified from 17 RefSeq proteomes, including unfiltered, filtered, clade-specific, and reference sequences, provided in six separate sheets:

- **Sheet 1 (All Identified PPRs):** List of all 19,352 PPR proteins identified across the 17 RefSeq proteomes, including taxonomy and sequence information.
- **Sheet 2 (Filtered Unique PPRs):** Subset of unique PPR proteins after filtering.
- **Sheet 3 (siRNA-PPR Clade):** PPR proteins belonging to the core siRNA-PPR phylogenetic clade.
- **Sheet 4 (siRNA-PPR Para-clade):** PPR proteins belonging to the siRNA-PPR para-clade.
- **Sheet 5 (Reference siRNA-PPRs):** Details of 168 reference siRNA-PPRs, including transcript ID, peptide ID, type, and amino acid sequences.
- **Sheet 6 (Clade siRNA-PPRs):** Details of 122 siRNA-PPRs within the phylogenetic clade.

Supplementary Data 9

Title: Analysis of siRNA-producing PPRs, predicted miRNA-PPR target interactions, and clade proportions in soybean.

Legend: Comprehensive dataset for siRNA-producing PPR loci and small RNA regulatory interactions in soybean (Glycine max), provided in four separate sheets:

- **Sheet 1 (PPR-siRNA Abundance):** Normalized abundance of PPR-siRNAs in soybean leaves.
- **Sheet 2 (psRNATarget Interactions):** Predicted miRNA-PPR target interactions identified by psRNATarget in soybean.
- **Sheet 3 (GSTAr Interactions):** Predicted miRNA-PPR target interactions identified by GSTAr in soybean.
- **Sheet 4 (Clade Proportions):** Proportion of PPR-siRNAs produced from individual phylogenetic clades in soybean.

Supplementary Data 10

Title: Oligonucleotides used in this study.

Legend: List of oligonucleotides and primers used in this study, including sequence information and corresponding applications (qPCR for Phytophthora and Arabidopsis genes, stem-loop qRT-PCR, P. capsici biomass quantification, and vector construction).
